# Adaptation of redox metabolism in drug-tolerant persister cells is a vulnerability to prevent relapse in pancreatic cancer

**DOI:** 10.1038/s41389-025-00591-0

**Published:** 2025-12-09

**Authors:** Nadine Abdel Hadi, Gabriela Reyes-Castellanos, Tristan Gicquel, Scarlett Gallardo-Arriaga, Emma Cosialls, Emeline Boet, Jean-Emmanuel Sarry, Rawand Masoud, Juan Iovanna, Alice Carrier

**Affiliations:** 1 Aix Marseille Univ, Inserm, CNRS, Institut Paoli-Calmettes, Centre de Recherche en Cancérologie de Marseille (CRCM), Marseille, France; 2 Centre de Recherches en Cancérologie de Toulouse, Université de Toulouse, Inserm CNRS, Toulouse, France

**Keywords:** Cancer metabolism, Target identification

## Abstract

Pancreatic Ductal Adenocarcinoma (PDAC) remains a major unresolved disease because of its remarkable therapeutic resistance. Even patients who respond to initial therapy experience relapse in most cases. The mechanisms underlying therapy-acquired resistance supporting relapse are poorly understood. In this study, we aimed to determine the metabolic features of PDAC during relapse, specifically adaptations of mitochondrial oxidative metabolism. We used preclinical PDAC mouse models (patient-derived xenografts and murine syngeneic allografts) that present regression under initial chemotherapeutic treatment but relapse after a certain time. Relapsed tumors were analyzed ex vivo by flow cytometry to measure mitochondrial and redox characteristics. Molecular mechanisms were investigated by quantification of ATP and antioxidants levels, bulk RNA-sequencing and RT-qPCR. We show increased mitochondrial mass, ATP levels, mitochondrial superoxide anions, and total ROS levels, in relapsed compared to control tumors in both models; mitochondrial membrane potential is increased in the xenografts model only. These metabolic features are also observed in tumors during treatment-induced regression and at relapse onset. At the molecular level, antioxidant defenses are increased in relapsed tumors and during treatment. These data suggest that metabolic adaptations occurring during treatment-induced regression may favor the survival of drug-tolerant persister (DTP) cells, which persist during the subsequent minimal residual disease and are responsible for cancer relapse. Finally, the combined treatment of arsenic trioxide (ROS inducer) and buthionine sulfoximine (glutathione synthesis inhibitor) is able to completely prevent relapse in PDAC xenografts. In conclusion, redox metabolism is a vulnerability of pancreatic DTP cancer cells that can be targeted to prevent relapse.

## Introduction

Pancreatic Ductal Adenocarcinoma (PDAC) is the most common cancer of the pancreas. Pancreatic cancer is the third leading cause of cancer-related deaths in the Western world and the sixth worldwide in men and women combined [1, 2], and is predicted to become the second in the USA by 2026 [3]. It remains an incurable disease, with the lowest 5-year survival rate after diagnosis of only 13% [2]. This poor prognosis is linked to lack of early diagnosis with high percentage of locally advanced or metastatic diseases at the time of diagnosis, and the limited effectiveness of anti-tumor treatments, including immunotherapies [4–7]. A small proportion of patients (less than 20%) show a localized tumor eligible for resection at diagnosis. Surgery followed by chemotherapy increases the median survival from 6 to 21 months, but most of the patients experience treatment resistance and develop disease recurrence at some point during treatment or after remission [8, 9]. It is therefore imperative to identify more effective therapies against this terrible disease, which requires a better understanding of the mechanisms of resistance to therapy.

Primary resistance is an intrinsic non-response to conventional treatments, while acquired resistance is therapy-induced resistance driving relapse (recurrence) after an initial response to treatment [10, 11]. Regarding acquired resistance, a major role has recently been shown for a subpopulation of drug-tolerant persister (DTP) cancer cells, which survive anti-cancer agent treatment through different strategies of adaptation, and are able to resume proliferation thus from which relapsed tumors emerge [12–15]. A common feature of many DTP cancer cells is increased mitochondrial oxidative metabolism, which has been demonstrated in different cancer types [16–21]. Another metabolic characteristic of these cells is the overproduction of mitochondrial reactive oxygen species (ROS) and the increase of antioxidant defenses to counteract them, thus allowing cell survival [22]. Such a process includes the increase in glutathione levels, which is commonly observed in various DTP cancer cells [23–25]. In addition, phospholipid glutathione peroxidase 4 (GPX4) activity has been shown to increase in DTP cells from melanoma and breast, lung, and colorectal cancer [24, 26, 27].

In PDAC, the importance of mitochondria in therapeutic resistance was suggested ten years ago by two pioneer studies showing the dependence of dormant/cancer stem cells (CSC) to OXPHOS [28, 29]. In our laboratory, we assessed mitochondrial metabolism in bulk tumors (not specifically the CSC), and we first showed heterogeneity between PDAC cells from different patients for the two main energy-producing pathways, glycolysis and mitochondrial oxidative phosphorylation (OXPHOS) [30]. We then found that the chemosensitivity of high OXPHOS tumors can be increased by inhibiting mitochondrial respiratory complex I with phenformin in vitro and in vivo [30, 31]. Moreover, we showed that mitochondrial respiration is dependent on fatty acid oxidation (FAO) in all PDAC tumors, and unveiled a synergy between chemotherapy and perhexiline (known as a FAO inhibitor) cytotoxicity [32]. Importantly, we found that an in vivo treatment combining perhexiline with standard chemotherapy, gemcitabine, was able to induce complete regression in one patient-derived xenograft (PDX) model (PDAC084T) among three that were gemcitabine-resistant [32]. Of note, complete regression was obtained for a fourth PDX model (PDAC032T) when treated with gemcitabine alone. Collectively, our previous results demonstrate that OXPHOS is a vulnerability of a subset of PDAC tumors that can be targeted to overcome primary resistance to therapy. Besides, OXPHOS was also shown to be involved in pancreatic cancer progression by promoting stemness and metastasis formation [33–38].

In our study published in 2023 [32], we extended the experiment by allowing the mice of the two PDX models (PDAC032T and PDAC084T) whose tumors had regressed during chemotherapy to live, and we observed relentless tumor regrowth after a certain period of time, named the minimal residual disease (MRD). Importantly, the relapsed tumors were shown to respond to a second cycle of treatment [32], suggesting that they emerge from residual drug-tolerant cancer cells that survived during the first cycle of treatment. Thus, these two PDX models provided opportunities to study the therapy-induced acquired tolerance in PDAC.

Based on the above data, in the present study, we explored mitochondrial metabolic adaptations supporting PDAC tumor recurrence, in particular, adaptations in redox metabolism. This study was conducted exclusively in vivo and ex vivo using our preclinical mouse models (PDX and syngeneic allografts) that relapse after chemotherapy-induced regression.

## Materials and methods

### Animal models

#### Xenograft mouse models of PDAC

We used the two PDAC patient-derived xenograft (PDX) xenograft models of relapse that we described in Reyes-Castellanos et al. [32], namely PDAC032T and PDAC084T, which always relapse after cancer remission induced by the chemotherapy, i.e., gemcitabine alone or combined with perhexiline, respectively. Recipient mice were 5–6-week-old athymic female, Swiss nude mice, SOPF (Specific and Opportunistic Pathogen Free) health status strain Crl:Nu(lco)-Foxn1^nu^ (Charles River, France). To obtain the xenografted mice, the subcutaneous tumor from an initial mouse donor was removed and finely minced with a scalpel. Then, 150 mg of tumor’s pieces were mixed with 50 μl of Matrigel and implanted with a trocar (10 Gauge) in the subcutaneous space of recipient isoflurane-anesthetized mice. A cohort of 15 recipient mice, on average, was generated with the same donor mouse tumor, thus limiting the variability of results within one experiment. Tumor volume was measured twice per week using a digital caliper, and tumor volume was calculated using the formula *V* = Length × (width)^2^/2.

When xenografts reached ~200 mm^3^ volume, mice were randomly assigned in a treatment group in which the average of all tumors was 200 mm^3^. Treatments were administered by intraperitoneal (IP) injection during 1 month as follows: gemcitabine 120 mg/kg IP twice a week for the PDX PDAC032T, or gemcitabine plus perhexiline (120 mg/kg IP twice a week and 5 mg/kg IP every other day, respectively) for the PDAC084T PDX. Gemcitabine (Gemzar) was provided by Eli Lilly & Co., and perhexiline maleate salt by Sigma-Aldrich. Vehicle-injected mice (controls) were injected with PBS in the case of gemcitabine controls or 3% DMSO in PBS for combination treatment controls. Mice whose tumor volume reached 1.5 cm^3^ were ethically sacrificed and tumors removed. Treatment to induce tumor regression is limited to a duration of 1 month, which is sufficient to lead to remission. Then, treatment is stopped, and the mice are left alive to monitor their relapse.

The in vivo relapse-preventing effect of arsenic trioxide (ATO, AS_2_O_3_, Sigma-Aldrich) and L-buthionine sulfoximine (BSO, Sigma-Aldrich) was evaluated in the xenograft mouse model. Mice were divided into different groups (at least *n* = 4 per condition): PBS (vehicle control), AS_2_O_3_ (0.2 mg/kg), and AS_2_O_3_ (0.2 mg/kg) + BSO (0.3 mg/kg). AS_2_O_3_ was administrated IP daily (5 days a week), starting right after the end of the 1-month chemotherapy treatment, inducing complete tumor regression, and during one, two, or three months. BSO was administrated IP 3 times per week for 2 months, starting 9 and 16 days (for PDAC032T and PDAC084T xenografts, respectively) after the end of the 1-month chemotherapy treatment and ATO start of treatment, for the sake of animal welfare. The use of low doses, even though both molecules are non-toxic in vivo, is based on the literature [39–41]. Treatment with BSO alone was not tested, as it has never shown antitumor action as a single treatment [41, 42] and also because of the limitation in the number of mice xenografted with the same donor mouse tumor.

#### Syngeneic allograft mouse model of PDAC

Orthotopic syngeneic allografts were generated as previously described [30]. The murine KPCluc2 cells were cultured in RPMI medium supplemented with 10% fetal bovine serum (FBS) and 600 μg/ml Hygromycine B (ThermoFisher Scientific) for selection of cells containing the vector encoding luciferase and GFP proteins, at 37 °C with 5% CO_2_ in a humidified atmosphere. One million KPCluc2 cells were IP injected into 5- to 6-week-old C57BL/6 female mice (immunocompetent strain, SOPF health status, Charles River, France). Tumoral growth was followed by bioluminescence upon injection of 3 mg luciferin-EF (Promega) using a Photon Imager device (Biospace Lab). Twelve days post-grafting, tumor-bearing mice were randomly assigned to two treatment cohorts (at least *n* = 5 per condition): vehicle control (treated with PBS), and gemcitabine 120 mg/kg IP twice a week during one month. Mice were sacrificed when they reached the ethical limit point.

All mice were kept under specific pathogen-free conditions and according to the current European regulation; the experimental protocol was approved by the Institutional Animal Care and Use Committee (#16711).

### Tumor dissociation

Xenograft and allograft tumors were dissociated using the gentle MACS™ Octo Dissociator with Heaters and the Tumor Dissociation Kit, mouse (130-096-730), as per manufacturer’s instructions (Miltenyi Biotec). Briefly, tumors were cut into 2–4 mm^3^ pieces and resuspended in RPMI 1640 medium supplemented with 2% FBS (Thermo Scientific, Waltham, MA, USA). These pieces then underwent mechanical and enzymatic digestion for 1 h. Immediately following dissociation, all single-cell suspensions were filtered using a MACS SmartStrainer (70 µm). The suspensions were centrifuged for 7 min at 300 × *g* at room temperature, the supernatant was aspirated, and the cells were resuspended in RPMI 2% FBS medium. Cells were counted by flow cytometry using the MACSQuant-VYB cell cytometer (Miltenyi Biotec).

### Flow cytometry ex vivo

All metabolic fluorescent probes were purchased from Invitrogen^TM^.

#### Mitochondrial mass and mitochondrial membrane potential measurements

Mitochondrial mass and mitochondrial membrane potential measurements were performed using MitoTracker Deep Red (M22426) and the MitoProbe™ TMRM Kit (M20036), respectively. Briefly, 200,000 cells were collected after tumor dissociation, centrifuged, and labeled with MitoTracker or TMRM to a final concentration of 200 nM and 20 nM in PBS at 37 °C for 10 min and 30 min, respectively. Then, 10,000 events per sample were acquired in a MACSQuant-VYB cytometer (Miltenyi Biotec), and data analysis was performed using the FlowJo software.

#### Total ROS and mitochondrial superoxide anions detection

Total ROS and mitochondrial superoxide anions measurements were performed using CellROX Orange (C-10443) and MitoSOX Red (M36008), respectively. Briefly, after tumor dissociation, 200,000 cells were collected, centrifuged, and labeled with CellROX or MitoSOX to a final concentration of 5 µM and 10 µM (in PBS for MitoSOX, and culture medium for CellROX) for 30 min and 20 min at 37 °C, respectively. After incubation, cells were centrifuged and resuspended in PBS 1x for flow cytometry analysis. 10,000 events per sample were acquired in a MACSQuant-VYB cytometer (Miltenyi Biotec), and data analysis was performed using the FlowJo software.

### ATP level measurement ex vivo

Total ATP level was measured using the cell viability assay (Cell-Titer Glo Kit; Promega) according to the manufacturer’s instructions. Immediately after tumor dissociation, 50,000 cells from each single-cell suspension were resuspended in 100 µl of RPMI 2% FBS medium and distributed in four replicates in a 96-well flat-bottom culture plate. Cells were then treated with PBS (Control), 1 µM oligomycin (inhibitor of mitochondrial respiration), or 100 mM 2-DG (inhibitor of glycolysis). Following 1 hour of incubation at 37 °C, 100 µl of Cell Titer Glo reaction mix solution was added to each well for a final volume of 200 µl. Plates were then analyzed by luminescence using Tristar LB 941 apparatus (Berthold Technologies). The background relative light unit (RLU) was subtracted from each RLU value. By comparing the different conditions, total ATP (PBS condition) and percentages of both mitochondrial and glycolytic ATP were determined: mitochondrial ATP = Total ATP - ATP (oligomycin); mitochondrial ATP (%) = mitochondrial ATP/Total ATP *100; glycolytic ATP = Total ATP - ATP (2DG); glycolytic ATP (%) = glycolytic ATP/Total ATP *100.

### Quantification of small antioxidant molecules

#### GSH/GSSG measurement

GSH/GSSG-Glo Assay kit (Promega, V6611) was used following manufacturer’s protocol with some modifications. Briefly, 10 mg of tumor tissue was crushed using a Precellys® Evolution device, resuspended in 50 µl of PBS/EDTA, and distributed in a 96-well plate. After homogenization, 50 μl of Total Glutathione Lysis Reagent (for Total glutathione measurement) or Oxidized Glutathione Lysis Reagent (for GSSG measurement) was added. Luciferin Generation Reagent and Detection Reagent were added to all wells, and luminescence was recorded using Tristar LB 941 apparatus (Berthold Technologies). By comparing the different conditions, GSH/GSSG ratio was calculated using the following equation: GSH/GSSG = (total glutathione RLU - GSSG RLU)/(GSSG RLU/2).

#### NADPH measurement

Ten micrograms of tumor tissue were crushed using a Precellys® Evolution, resuspended in 50 µl of PBS/EDTA, and distributed in a 96-well plate. Measurement was performed according to the manufacturer’s protocol. Briefly, 50 µl of NADP/NADPH-Glo™ Detection Reagent (Promega, G9081) was added to each well and incubated for 30 min at RT. Luminescence was recorded using Tristar LB 941 apparatus (Berthold Technologies).

### Gene expression analysis by RT-qPCR

Total RNA was isolated from 20 to 25 mg of tumor piece using both TRIzol (Invitrogen) and the Qiagen total RNA isolation kit (Qiagen, ref 74004) to avoid protein and extracellular matrix accumulation in the columns. The tumor piece was lysed in 600 µl RLT Buffer (from Qiagen kit) with 1% β-Mercaptoethanol using beads tube from Precellys lysing kit for hard tissue (ref P000917-LYSK0-A, 3x Cycle 1500 rpm, 15 s of mix, 10 s of rest) and directly centrifuged 3 min at 10,000 × *g* at RT. Supernatant was transferred in 400 µl of TRIzol and incubated for 5 min at RT followed by addition of 150 µl of chloroforme. Tubes were carefully mixed and incubated 3 min at RT before centrifugation (12,000 × *g*, 5 min, 4 °C). The transparent upper phase containing RNAs was transferred in a tube containing 500 µl of 70% ethanol, mixed, and transferred inside Qiagen RNA isolation kit columns according to manufacturer’s instructions, which were followed until the end of extraction. RNA samples were subjected to reverse-transcription (RT) using the Go Script reagent (Promega) following manufacturer’s instructions. Next, Real-Time quantitative PCR was performed in triplicate using Takara reagents and the Stratagene cycler Mx3005P QPCR System. Raw values were normalized with the housekeeping gene TBP1 for the same cDNA sample. We used the human primers for Nrf2, HO-1, SLC7A11, and GPX4, involved in the antioxidant defense, and PGC-1α and TFAM, involved in mitochondrial homeostasis. Primer sequences can be found in [43]. For each RNA sample, RT reaction was done twice to generate 2 batches of cDNA, which were amplified by qPCR in three independent experiments for each gene.

### Transcriptomic analysis by bulk RNA sequencing

#### Total RNA isolation from tumors

RNA was extracted from PDAC xenografts using the RNeasy Mini kit (Qiagen) as described above. RNA integrity and concentration were assessed using the Agilent 2100 Bioanalyzer (Agilent Technologies, Palo Alto, CA). The average RIN (RNA integrity number) values for all samples were comprised between 9.3 and 10, ensuring a high quality of isolated RNAs.

#### Bulk RNA sequencing

The preparation of mRNA libraries was realized following manufacturer’s recommendations (kapa mRNA HyperPrep from ROCHE). Final samples pooled library prep were sequenced on ILLUMINA Novaseq 6000 with S1-200cycles cartridge (2 × 1600Millions of 100-base reads), corresponding to 2 × 30Millions of reads per sample after demultiplexing.

#### Bulk RNA sequencing data analysis

Quality control has been performed on the fastq files using FastQC (v0.11.9) (http://www.bioinformatics.babraham.ac.uk/projects/fastqc). To map the sequenced reads to the human reference genome, we made use of STAR (v2.7.3a). From these mapped reads, gene counts were then quantified using featureCounts (v2.0.1). Starting from the raw gene counts, normalization and differential expression analysis have then been performed using DESeq2 (v 1.22.2).

#### Differential gene expression analysis

Differential expression analysis was performed using the **limma** package (R/Bioconductor). Genes with a log₂ fold change (logFC) > 1.25 and an adjusted *p* value < 0.05 were considered significantly differentially expressed. A total of 688 upregulated genes were identified in the PDAC032T relapse group compared to untreated.

#### Functional enrichment analysis

Functional enrichment analysis of the upregulated gene set was conducted in R using the following tools: **enrichGO** (from the clusterProfiler package) for Gene Ontology (GO) biological processes, **enrichKEGG** for KEGG pathway analysis, **enrichR** for Reactome. Default parameters were used for all enrichment tools. Significantly enriched terms and pathways were defined as those with an adjusted *p* value < 0.05.

#### Gene set enrichment analysis (GSEA)

Gene set enrichment analysis (GSEA) was performed using GSEA v4.1 tool (Broad Institute). Following parameters were used: Number of permutations = 1000, permutation type = gene set, Chip = Human_Ensembl_Gene_ID_MSigDB.v2022.1.Hs. chip. Other parameters were left at default values. The normalized enrichment scores (NES) were computed from PDAC032T relapse group compared to PDAC032T control group. Genes with FDR *q* value < 0.05 and fold change ≥ 1.5 were considered of interest.

### In vitro assays

#### Cell viability

The human PDAC032T and PDAC084T primary PDAC cells were cultured in serum-free ductal media (SFDM) at 37 °C with 5% CO_2_ in a humidified atmosphere as reported previously [30, 32]. SFDM is a complex medium supporting the PDAC primary cell growth and containing DMEM-F12, nicotinamide, glucose, hormones, growth factors, and Nu-serum, providing a low-protein alternative to FBS. Cells were seeded in 96-well plates in triplicate (5000 cells per well) and the corresponding treatment was administered the day after. Cells were treated with perhexiline (7 µM), gemcitabine (1 µM), or the combination for 24 h. These treatments were done in the presence and absence of the antioxidant N-acetylcysteine (NAC, Sigma-Aldrich) at 2.5 mM. Next, cell viability was determined by the Crystal violet viability assay, which is independent of cell metabolism. For this, cells were fixed in glutaraldehyde (1%), washed twice with PBS, stained with Crystal violet (0.1%) for 10 min, and then washed three times with PBS. Crystals were solubilized in SDS (1%), and absorbance was measured at 600 nm using an Epoch-Biotek spectrophotometer.

#### Total ROS measurement by flow cytometry

Cells were seeded in 12-well plates in duplicates (200,000 cells/1 ml medium/well) and the day after, treatments were administered. Cells were treated with perhexiline (7 µM), gemcitabine (1 µM), or the combination for 24 h. After treatments, the medium was supplemented with CellROX Orange at a final concentration of 5 μM. Cells were incubated for 30 min at 37 °C, then harvested with pre-warmed Accutase (Gibco) and resuspended in PBS for flow cytometry analysis. 10,000 events per sample were acquired in a MACSQuant-VYB cytometer (Miltenyi Biotec), and data analysis was done with the FlowJo software.

### Immunoblotting

Tumor pieces were lyzed using Precellys Lysing Kit (MK28R Hard Tissue 2 mL, Bertin Technologies). Each tumor sample was homogenized on ice in 400 µl of RIPA Buffer (supplemented with Protease and Phosphatase Inhibitor) using the Precellys Evolution with the Hard Tissue Programme. Protein concentrations were determined with the Pierce BCA protein assay kit (23225, Thermo Fisher Scientific). Equal protein amounts (35 μg) diluted in a 4× Laemmli buffer were denatured by heating at 95 °C for 5 min and separated by electrophoresis on 4–20% Mini-PROTEAN TGX Precast Protein Gel, then transferred onto a 0.45μm nitrocellulose membrane. All membranes were systematically stained with Ponceau red to confirm equal protein loading and transfer. Membranes were blocked with 5% non-fat dry milk in TBS-T (TBS with 0.1% Tween-20) for 1 h at room temperature and then incubated in 5% BSA in TBS-T with appropriate primary antibodies at 4 °C overnight: HO-1 (1:2000, Abnova - A303-662A), FTH (1:1000, abcam 75973), and GPX4 (1:1000, Abcam 125066). Membranes were then washed three times with TBS-T, and incubated with the appropriate HRP-coupled secondary antibody at 1:2000 for 1h30min at RT, before being revealed with ECL (Immobilion Western ECL, Millipore). Signal detection was performed using Fusion FX7 imagine system. Protein band intensities (Raw Integrated Density) were quantified using ImageJ Software after background subtraction (rolling ball radius: 50 pixels). Signal of proteins of interest was normalized to Ponceau Red to account for loading variation.

### Statistical analyses

Results are expressed as the mean ± SD of duplicates or triplicates, except for in vitro cell viability data shown in mean ± SEM, and at least two or three independent experiments were done for each analysis. Statistical analysis of data was performed with GraphPad Prism 8 (GraphPad Software) using two-tailed unpaired Student’s *t*-test. *P* values < 0.05 were considered to be statistically significant (*P < 0.05, **P < 0.01, ***P < 0.001, and ****P < 0.0001).

## Results

### The mitochondrial metabolism is reprogrammed in relapsed PDAC tumors

**Fig. 1:**
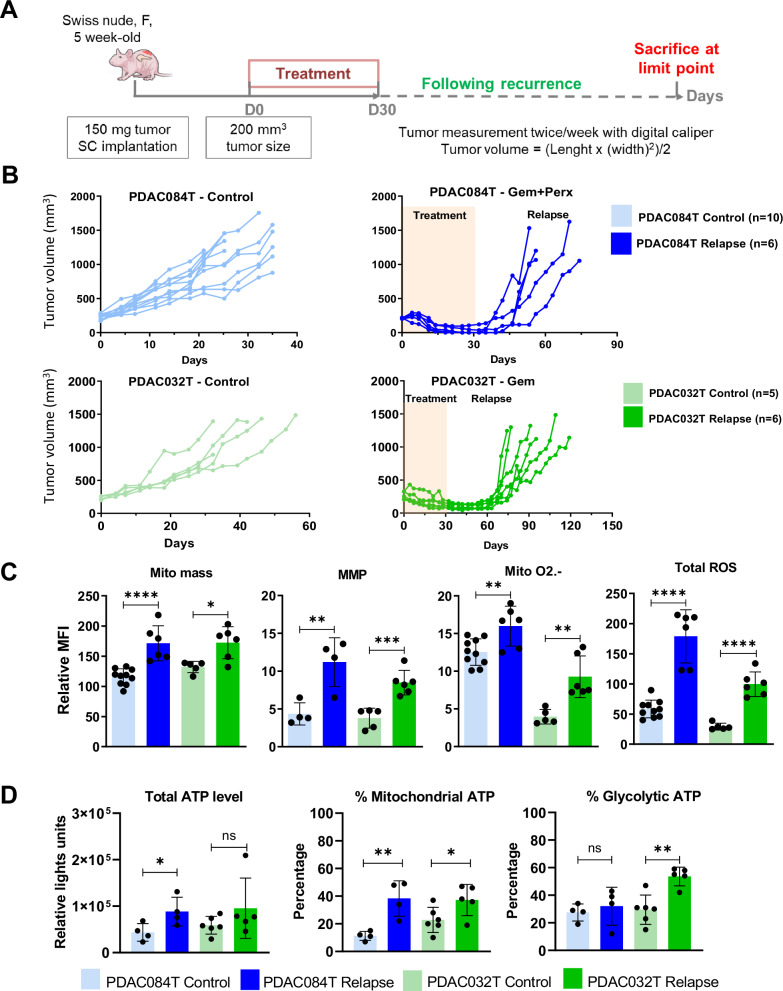
Mitochondrial and redox metabolisms are reprogrammed in relapsed PDAC xenografts. **A** Schematic of experimental protocol. Pieces of tumor from PDX were implanted in the subcutaneous space of recipient female Swiss nude mice. When tumors reached 200 mm^3^ volume, mice were assigned to treatment groups and treated for one month (30 days), then the treatment was stopped and the recurrence was followed. Mice were sacrificed according to the ethical limit point. **B** Tumor volume from start of 1-month treatment in the two different PDAC PDX relapse models, PDAC084T treated with the combination gemcitabine plus perhexiline (Gem+Perx) and PDAC032T treated with gemcitabine (Gem), and untreated controls. PDAC xenografts always relapse after complete regression induced by chemotherapy. **C** Mitochondrial mass (Mito mass), mitochondrial membrane potential (MMP), mitochondrial superoxide anions (Mito O_2_^.^^−^), and total reactive oxygen species (Total ROS) levels were measured by flow cytometry with the MitoTracker Deep Red, TMRM, MitoSOX red, and CellROX orange fluorescent probes, respectively, in relapsed tumors at ethical limit point. Median fluorescence intensity (MFI) is shown relative to that of unlabeled cells. Each dot corresponds to one mouse, and the bars show the mean ± SD. **D** Total ATP level and percentages of mitochondrial and glycolytic ATP were measured using the Cell viability assay (Cell-Titer Glo Kit). For this, specific inhibitors were used: oligomycin (1 µM) or 2-DG (100 mM) to determine percentages of mitochondrial and glycolytic ATP, respectively. Unpaired student’s *t*-test was used for statistical analyzes comparing each group with its untreated control group. *, **, *** and **** correspond to *p* < 0.05, 0.01, 0.001, and 0.0001, respectively; ns non-significant difference.

To elucidate the involvement of mitochondria in therapy-acquired tolerance in PDAC, we took advantage of 2 models of relapse after chemotherapy-induced cancer remission generated in our previous work [32]. We used the two subcutaneous PDAC xenograft models, PDAC084T and PDAC032T, which always relapse after complete regression induced by the chemotherapy, namely gemcitabine combined with perhexiline (FAO inhibitor) or gemcitabine alone, respectively (Fig. 1A, B). We analyzed mitochondrial characteristics and ROS levels in relapsed tumors at the ethical end point ex vivo. Flow cytometry data presented in Fig. 1C and S1 illustrate that mitochondrial mass, mitochondrial membrane potential (MMP, reflecting respiratory activity), mitochondrial superoxide anion (O_2_^.−^) level, and total ROS level are higher in relapsed tumors (in both models) compared with controls (untreated). Interestingly, only a portion of the increase in MMP and mitochondrial O_2_^.−^ comes from the increase in mitochondrial mass, as shown in the normalized data (Fig. S2A). In addition, we found a higher level of total ATP in the relapsed PDAC084T xenograft, a higher percentage of ATP produced by mitochondria in both relapsed xenografts, and a higher percentage of ATP produced by glycolysis in the relapsed PDAC032T xenograft (Figs. 1D and S2B), suggesting higher energy production in both settings.

**Fig. 2:**
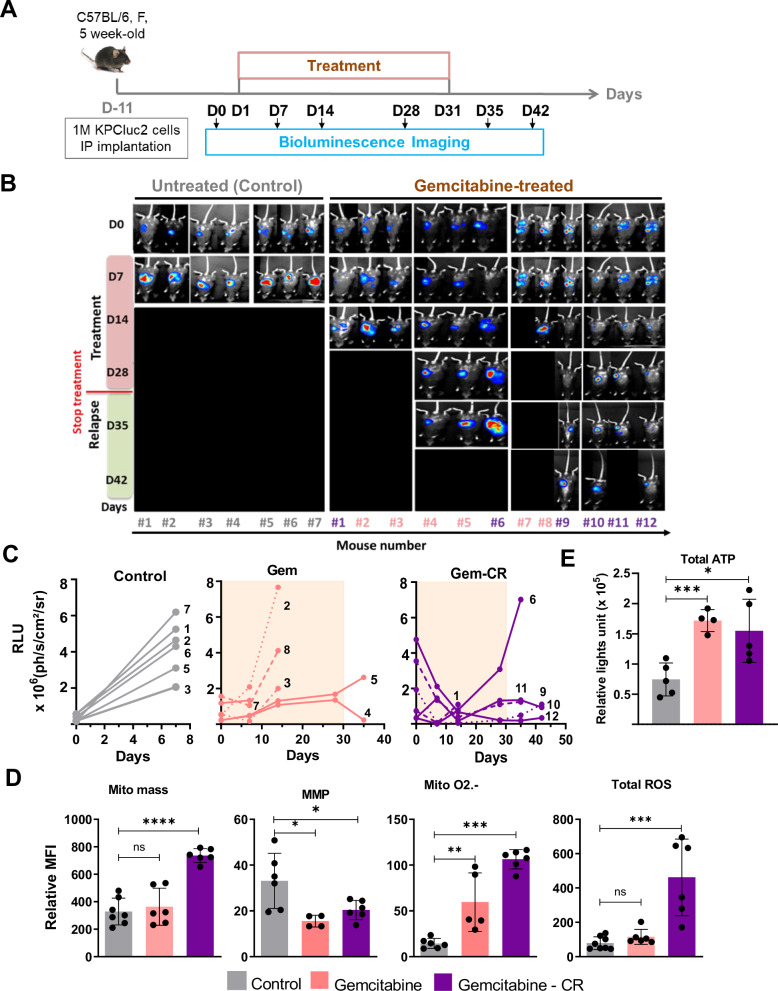
PDAC syngeneic allografts also show changes in mitochondrial and redox metabolisms after relapse. **A** Schematic of experimental protocol. Orthotopic syngeneic allografts were generated by intraperitoneal injection of one million of the murine KPCluc2 cells into 5-week-old female C57BL/6 immunocompetent mice. Tumoral growth was followed by bioluminescence using a Photon Imager device (Biospace Lab). Twelve days post-grafting, tumor-bearing mice were randomized to two treatment cohorts, vehicle control (PBS) and gemcitabine during one month. Mice were sacrificed according to the ethical limit point**. B**, **C** Longitudinal in vivo bioluminescence imaging analysis of KPCluc2 cell-grafted mice, untreated (Control) or treated with gemcitabine starting 12 days after implantation. **B** Bioluminescence images. **C** Bioluminescent signal quantification in which each line, solid or dotted, represents one mouse (RLU = relative light unit). Tumors in mice 2, 3, 4, 5, 7, and 8 responded to gemcitabine treatment but did not completely regress (Gem group); their numbers are shown in pink. In contrast, tumors in mice 1, 6, 9, 10, 11, and 12 regressed completely as evidenced by the absence or very low level of bioluminescence at at least one stage of treatment effect analysis (Gem-CR group); their numbers are shown in purple. The bioluminescent signal is expressed in photons per second per square centimeter per steradian (ph/s/cm^2^/sr). **D** Mitochondrial mass, MMP, Mitochondrial O2.- and total ROS level were measured in tumors as in Fig. 1. Median fluorescence intensity (MFI) is shown relative to unlabeled cells. Each dot corresponds to one mouse, and the bars show the mean ± SD. **E** Total ATP level was measured using the cell viability assay (Cell-Titer Glo Kit). Unpaired Student’s *T*-test was used for statistical analyzes comparing each group with that of the untreated. *, **, *** and **** correspond to *p* < 0.05, 0.01, 0.001, and 0.0001, respectively; ns non-significant difference.

As xenografts are made in immunodeficient mice, and the immune tumor microenvironment can influence relapse, we also investigated this question in an immunocompetent PDAC model, namely syngeneic allografts that we reported previously [30]. In this model, murine luciferase-expressing PDAC cells implanted into the peritoneal cavity of syngeneic recipient mice grow preferentially in the pancreas, thus providing a very convenient orthotopic allograft model in which tumor growth and regression can be monitored by bioluminescence (Fig. 2A, B). All tumors respond to gemcitabine treatment and most regress either completely or partially, and all relapse during or after the end of the 1-month treatment (Fig. 2B, C). As for the xenografts presented above, metabolic variations during relapse were also observed in this immunocompetent mouse model. Mitochondrial mass, mitochondrial O_2_^.−^ and total ROS levels are higher in relapsed tumors compared to controls (untreated), but the difference is significant only for tumors that have fully regressed for mitochondrial mass and total ROS level (Fig. 2D). In contrast to the xenografts, MMP is decreased in relapsed tumors compared to controls (Fig. 2D). Only part of the increase in mitochondrial O_2_^.−^ comes from the increase in mitochondrial mass, whereas the decrease in MMP is amplified when normalized by mitochondrial mass (Fig. S2C). Next, we found a higher level of total ATP cells from relapsed allograft (Fig. 2E), suggesting higher energy production during relapse.

Collectively, these data show increases in mitochondrial and redox metabolisms in tumors that have relapsed after full treatment-induced regression compared to non-treated tumors, suggesting metabolic adaptations induced by the chemotherapy.

### Mitochondrial metabolic adaptations occur during treatment-induced regression

**Fig. 3:**
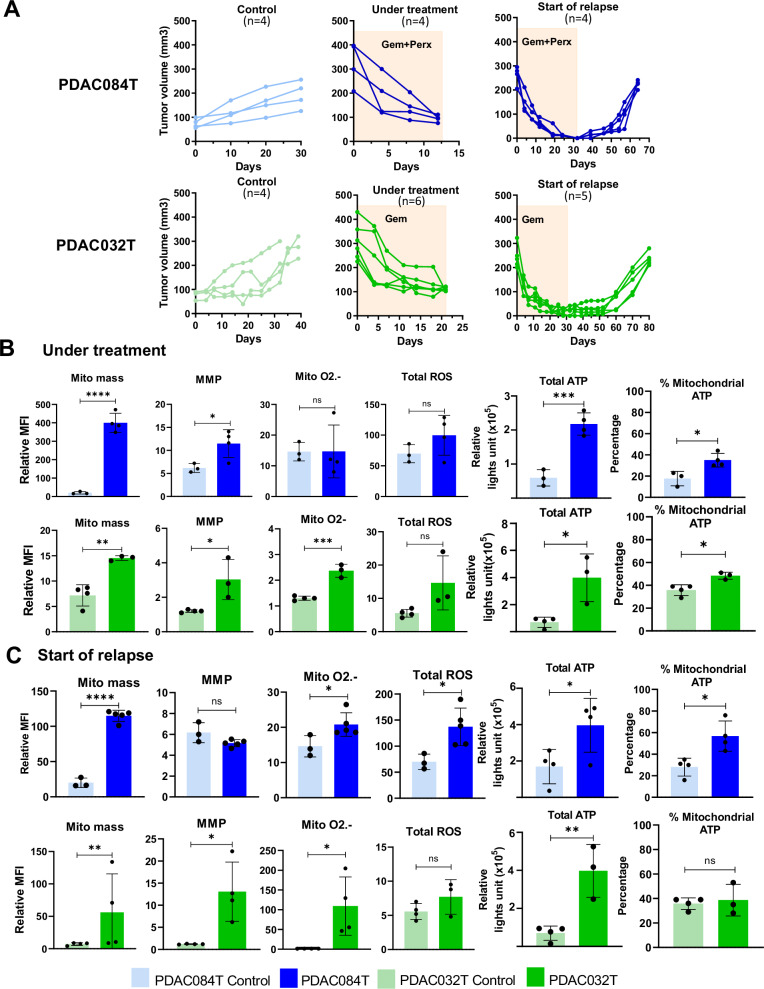
Mitochondrial metabolic reprogramming occurs during treatment-induced regression in PDAC xenografts. **A** Tumor volume in PDAC084T (Top), and PDAC032T (Bottom) during Gem+Perx combo or Gem alone treatment, respectively, before complete regression (“under treatment” context) and at the onset of relapse (“start of relapse” context), compared to untreated tumors (Control) which were analyzed at similar size (100–300 mm^3^). For the control groups, we show the measurement of tumor volumes from two weeks after inoculation. For the treated groups, we present the measurement of tumor volumes from the start of treatment, exactly as shown in Fig. 1. Mitochondrial mass, MMP, Mitochondrial O2.- and total ROS level were measured in tumors under treatment (**B**) and at the start of relapse (**C**). Total and mitochondrial ATP were measured in both context (**B**, **C**) using cell viability assay (Cell-Titer Glo Kit). Each dot corresponds to one mouse, and the bars show the mean ± SD. Significance was determined by Student’s *T*-test. *, **, *** and **** correspond to *p* < 0.05, 0.01, 0.001, and 0.0001, respectively; ns non-significant difference.

We wondered when metabolic changes occurred, in other words, whether they were related to tumor growth during relapse or whether they preexisted relapse. To answer this question, we analyzed xenografts during treatment before complete regression (“under treatment” setting) and at the onset of relapse (“start of relapse” setting), comparing them to untreated tumors of similar size (100–300 mm^3^) (Fig. 3A). We observed that most of the parameters analyzed are increased in both settings and in both xenograft models (Figs. 3B, C and S3), as observed in relapsed tumors at the end point. Importantly, mitochondrial mass is the parameter that was most increased, explaining the attenuation or even reversal of changes in MMP and mitochondrial superoxide after normalization with mitochondrial mass (Fig. S3A, B). These data suggest that the drug treatment, i.e., gemcitabine alone or combined with perhexiline for PDAC032T and PDAC084T xenografts, respectively, could promote the survival of treatment-adapted cells while inducing the death of most tumor cells by oxidative stress associated with the treatment. Indeed, in vitro treatments induce an increase in ROS level that can be prevented by supplementation with the antioxidant N-acetylcysteine (NAC), and a redox-induced loss of cell viability (Fig. S4).

Overall, these data show that chemotherapy treatment affects mitochondrial and redox metabolisms, and suggest that certain residual/persistent cells, now referred to as drug-tolerant persister (DTP) cancer cells, which survive through metabolic adaptations, are responsible for relapse in our in vivo PDAC experimental model.

### Antioxidant defenses are increased in relapsed tumors and in tumors during treatment

**Fig. 4:**
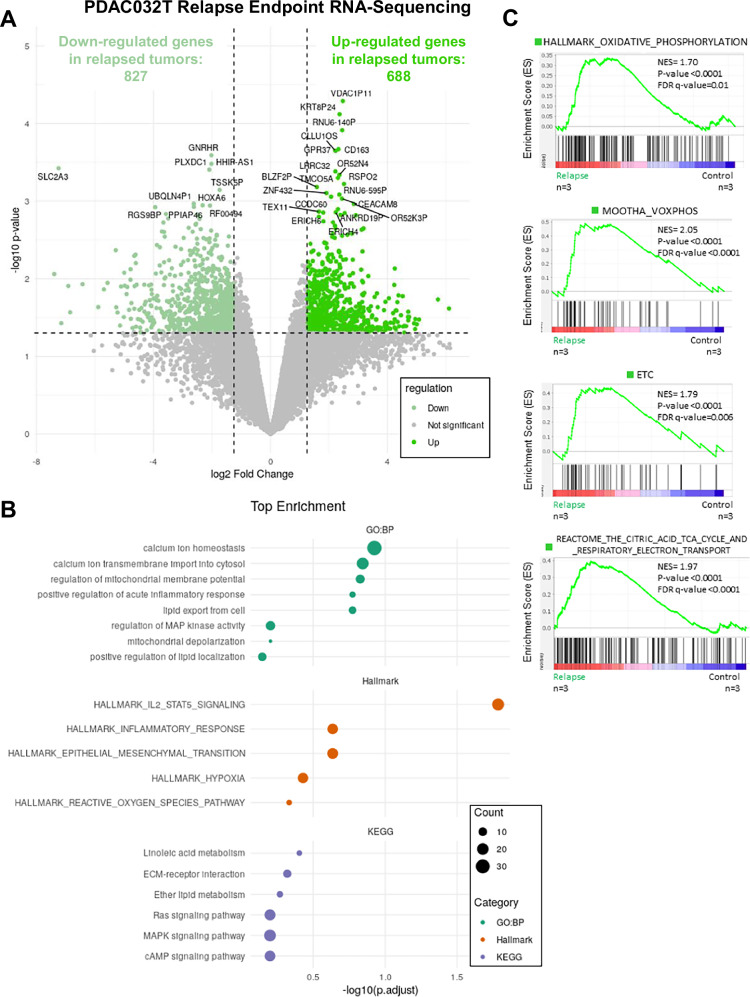
Transcriptomic analysis of relapsed PDAC032T xenografts by bulk RNA-sequencing reveals enrichment in mitochondrial and redox metabolic pathways. **A** Volcano plot of differentially expressed genes in PDAC032T xenografts at the relapse endpoint compared to untreated (*n* = 3 for each group). Genes with adjusted *P* < 0.05 are highlighted: light green indicates downregulated genes; dark green indicates upregulated genes. **B** Functional enrichment analysis of upregulated genes using enrichGO (for Gene Ontology biological processes), enrichKEGG (for KEGG pathways), and enrichR (for Reactome pathways). **C** Gene Set Enrichment Analysis (GSEA) of four mitochondrial gene signatures: Oxidative Phosphorylation, MOOTHA_VOXPHOS, ETC, and Reactome TCA Cycle and Respiratory Electron Transport. Significant enrichment was observed in relapsed PDAC032T tumors versus control. Top panels display normalized enrichment scores (NES); bottom panels show the ranking metric across the ranked gene list.

To go deeper into the molecular mechanisms underlying metabolic changes in PDAC relapsed tumors, we performed a transcriptomic study by bulk RNA-sequencing of PDAC032T relapsed tumors compared to untreated (controls), at relapse endpoint, as in the setting analyzed in Fig. 1 (*n* = 3 for each group). We found 688 upregulated and 827 downregulated genes (Fig. 4A). Functional enrichment analysis of the upregulated gene set was conducted for Gene Ontology (GO) biological processes, KEGG pathway analysis, and Reactome (Fig. 4B). These analyses unveil enrichment in metabolic pathways, namely mitochondrial (regulation of mitochondrial membrane potential, mitochondrial depolarization, and calcium ion homeostasis), lipid (lipid export from cell, positive regulation of lipid localization, linoleic acid metabolism, and ether lipid metabolism), and redox (Reactive Oxygen Species pathway) pathways. Enrichment in other pathways related to redox metabolism such as hypoxia and inflammatory response is also observed. Interestingly, these analyses also point to the epithelial-mesenchymal transition (EMT) pathway known to be upregulated in DTP cancer cells, involving redox signalling, and a major actor in cancer cell plasticity [22]. As partial EMT is associated with cancer cell plasticity and chemoresistance [44, 45], we applied Gene Set Enrichment Analysis (GSEA) on the RNA-seq data but did not observe any significant enrichment of the partial EMT signature after relapse (Fig. S5A).

To investigate further the mitochondrial pathways, we applied GSEA on the RNA-seq data and found that relapsed tumors positively correlate with changes in OXPHOS, MOOTHA_VOXPHOS, electron transport chain (ETC), and the citric acid (TCA) cycle gene signatures (Fig. 4C). We also looked at the expression of genes involved in mitochondrial mass homeostasis, and found increased expression (despite not statistically significant) of Peroxisome Proliferator-activated Receptor–γ Coactivator 1 alpha *PPARGC1A* encoding PGC-1α in relapsed tumors but not mitochondrial transcription factor A *TFAM* (Fig. S5B).

We also investigated further the redox pathway. A crucial metabolic adaptation observed in cancer cells during tumor development is the exacerbation of antioxidant defenses to cope with the increased production of ROS, which allows the survival of the adapted cells [46, 47]. This adaptation is also observed during oxidative stress induced by chemotherapy or radiotherapy [22, 48]. As metabolic reprogramming in DTP cancer cells promotes a robust antioxidant process [22], we assessed antioxidant defences in relapsed tumors. The main antioxidant defenses are antioxidant enzymes such as glutathione peroxidases (GPXs), superoxide dismutases, and catalase, as well as small antioxidant molecules such as glutathione and NADPH that are found to be abundant in cancer cells (schematic in Fig. S6A). In addition, the nuclear factor erythroid 2-related factor 2 (Nrf2) plays a crucial role in antioxidant gene expression; it can be controlled both at the level of expression and cellular localization since it is translocated to the nucleus only when ROS levels increase. One of the target genes of Nrf2 is *SLC7A11*, which encodes the cystine/glutamate transporter (xCT), which allows the entry of cystine into cells as a precursor of glutathione (tripeptide Glutamate-Cysteine-Glycine). Reduced glutathione (GSH) acts as a co-factor (electron donor) of the GPXs, whose antioxidant activity generates oxidized glutathione (GSSG) that is reduced by the glutathione reductase using NADPH as a co-factor.

**Fig. 5:**
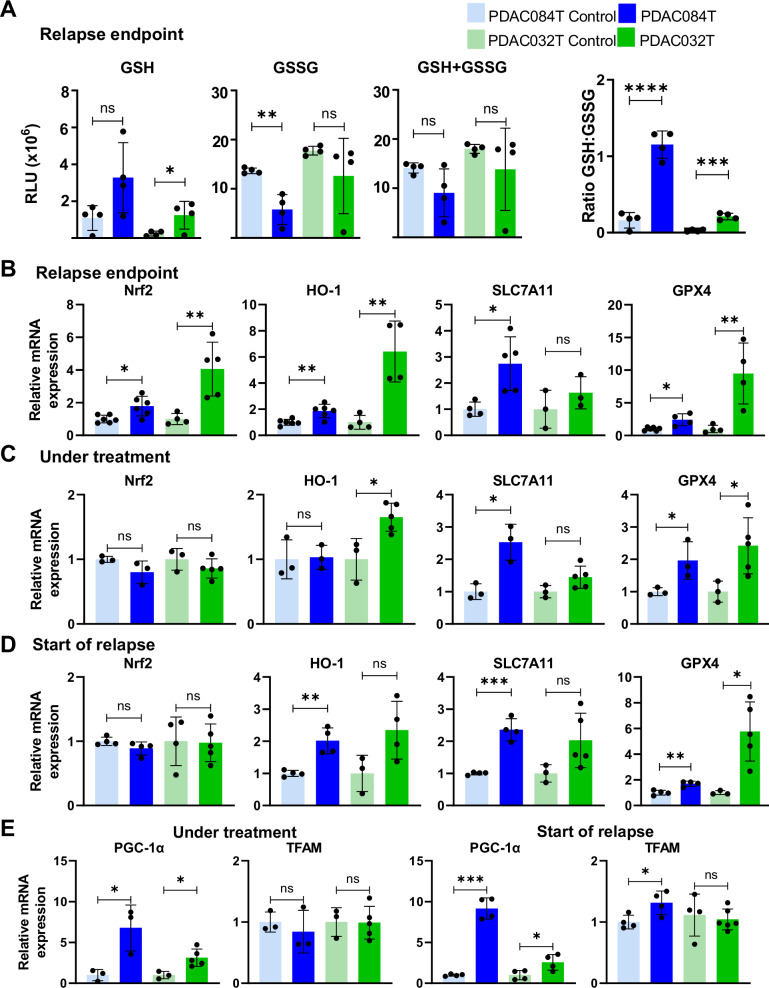
Antioxidant defenses are increased in relapsed tumors and in tumors during treatment. **A** Intracellular levels of reduced glutathione (GSH), oxidized glutathione (GSSG), and total glutathione (GSH + GSSG) were measured with the GSH/GSSG-Glo™ Assay (Promega kit) in PDAC relapsed tumors, and the ratio GSH/GSSG was calculated. RT-qPCR analysis of antioxidant gene expression: Nrf2, HO-1, SLC7A11, and GPX4, in relapsed PDAC tumors at endpoint (**B**), under treatment (**C**) and start of relapse (**D**). **E** The mRNA levels of PGC-1α and TFAM was monitored by RT-qPCR in PDAC tumors under treatment and start of relapse, respectively. Each dot corresponds to the mean of triplicates for one mouse, and the bars show the mean ± SD. Data are representative of three independent qPCR experiments. Significance was determined by unpaired Student’s *t* test. *, **, *** and **** correspond to *p* < 0.05, 0.01, 0.001, and 0.0001, respectively; ns non-significant difference.

We first quantified glutathione and NADPH levels in xenografts at relapse endpoint (Figs. 5A and S6B). We found increased GSH and NADP^+^ + NADPH levels in relapsed PDAC032T (not statistically significant for PDAC084T), decreased GSSG level in relapsed PDAC084T, and increased GSH/GSSG ratio and NADPH level in both xenograft models. These data demonstrate increased levels of the main small antioxidant molecules in relapsed tumors.

We then analyzed expression of several antioxidant genes by RT-qPCR. *Nrf2*, its targets *HO-1* and *SLC7A11*, and *GPX4* were found to be overexpressed in the relapse endpoint context (Fig. 5B). We also measured the expression of these genes in xenografts under treatment and at the onset of relapse (Fig. 5C, D). *Nrf2* was not found to be overexpressed in these settings. Its target gene *SLC7A11* was found to be overexpressed in the PDAC084T xenograft in both contexts. *HO-1* gene was found to be overexpressed in the PDAC084T xenograft at relapse onset, and in the PDAC032T xenograft under treatment. These data suggest that Nrf2 is activated even when not overexpressed. The *GPX4* gene was found overexpressed in both conditions. We also looked at the expression level of these genes in the RNA-seq data, and found only *GPX4* gene as over-expressed in relapsed endpoint tumors (Fig. S7A). We also found enrichment of the Nrf2 BIOCARTA_ARENRF2 pathway in relapsed tumors (Fig. S7B).

Finally, GPX4 and two Nrf2 targets, HO-1 and ferritin heavy chain (FTH), were analyzed at the proteomic level by immunoblotting in both xenograft models during treatment, revealing highly heterogeneous levels within each group, but an accumulation of FTH in tumors undergoing regression, unlike the other proteins tested (Fig. S7C).

RT-qPCR experiments were also performed to examine the expression of genes encoding proteins involved in mitochondrial homeostasis (PGC-1α and TFAM) and found *PGC-1α* overexpressed in xenografts under treatment and at the onset of relapse (Fig. 5E), and TFAM overexpressed only in PDAC084T tumors at relapse onset. These results suggest that increased mitochondrial mass could play a role in mitochondrial ROS overproduction.

Collectively, these data show that the PDAC DTP cells responsible for relapse have adapted to drug-induced oxidative stress by increasing antioxidant defenses.

### ROS accumulation is a vulnerability that can be targeted to prevent relapse

The property of cancer cells to have a high ROS content can be used therapeutically by further increasing the level of ROS to reach the threshold for induction of cell death [46, 47]. This was the course of action we took in an attempt to further increase the level of ROS in DTP cancer cells to prevent relapse. To this end, we started treatment with arsenic trioxide (ATO) just after the 1-month chemotherapeutic treatment leading to regression. ATO is an inhibitor of mitochondrial respiratory complex IV that is FDA approved for the treatment of acute promyelocytic leukemia [49, 50]. As a well-tolerated drug, it is being studied in other types of cancer, notably pancreatic cancer, where its action also involves induction of ROS generation [39, 41].

**Fig. 6:**
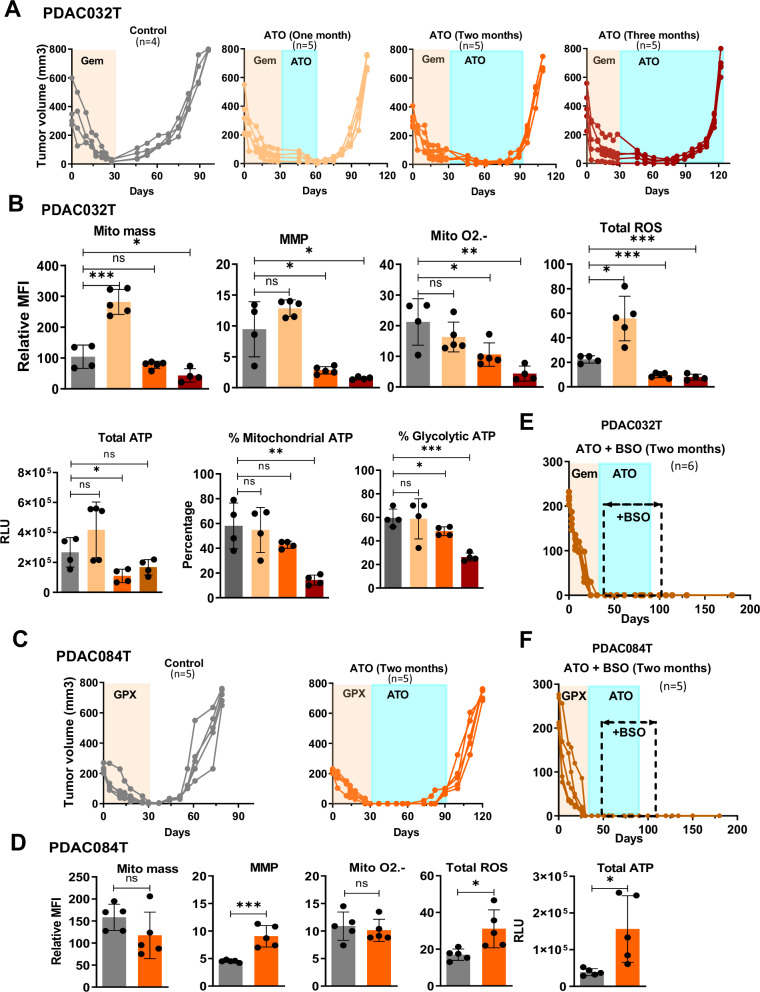
Targeting redox metabolism prevents relapse in both PDAC xenograft models. **A** Tumor volume in PDAC032T xenograft treated with ATO (0.2 mg/kg/day) for one, two or three months starting just after the end of the 1-month chemotherapy inducing complete regression. **B** Mitochondrial mass, MMP, Mitochondrial O2.- and total ROS level were measured in PDAC032T xenografts (Top). Median fluorescence intensity (MFI) is shown relative to unlabeled cells. Total ATP level, percentage of mitochondrial and glycolytic ATP were measured using the Cell viability assay (Cell-Titer Glo Kit) in PDAC032T tumor cells (Bottom). Specific inhibitors were used: oligomycin (1 µM) or 2-DG (100 mM) to determine mitochondrial and glycolytic ATP, respectively. **C** Tumor volume in PDAC084T xenograft treated with ATO (0.2 mg/kg daily) for two months starting after the end of combinatory treatment-induced complete regression. **D** Mitochondrial mass, MMP, Mitochondrial O2.- and total ROS level were measured in PDAC084T xenografts. Total ATP level was measured using the Cell viability assay (Cell-Titer Glo Kit). Tumor volume in PDAC032T (**E**) and PDAC084T **F** xenografts, respectively, treated with ATO (0.2 mg/kg daily) and BSO (0.3 mg/kg 3 times per week) during two months after the chemotherapy-induced complete regression. Unpaired *T*-test was used for statistical analyzes comparing each group with the untreated group. *, ** and *** *p* < 0.05, 0.01, 0.001, respectively; ns non-significant difference.

ATO treatment was first performed in PDAC032T xenograft model for 1, 2, or 3 months (Fig. 6A). We observed that ATO was able to delay relapse compared with control (relapse of non-ATO-treated mice) in the case of mice treated with ATO for 1 month. Tumor growth started to resume before the end of treatment in the case of mice treated with ATO for 2 months and 3 months. We analyzed mitochondrial characteristics and ROS level in recurrent tumors and found increased mitochondrial mass, ROS level, and MMP (despite this latter didn’t reach the statistically significance) in 1-month ATO-treated recurrent tumors compared with the control mice (Fig. 6B). In contrast, most of the analyzed metabolic characteristics were found to be decreased in recurrent ATO-treated tumors treated for 2 or 3 months compared with the control group. Finally, mitochondrial MMP and superoxide anion levels decreased in ATO-treated recurrent tumors when normalized by mitochondrial mass for some time points (Fig. S8A), as well as ATP level (Fig. 6B), suggesting an ATO-induced energy crisis consistent with its role in inhibiting mitochondrial respiration.

In addition, a 2-month ATO treatment was performed in PDAC084T xenograft model (Fig. 6C). We observed that ATO treatment delayed relapse compared with control (relapse of non-ATO-treated control mice). Tumor growth started to resume before the end of treatment, suggesting escape from ATO treatment, as observed for the PDAC032T xenograft model. We analyzed mitochondrial characteristics and ROS level in relapsed tumors, and found increased MMP and ROS level as well as ATP level compared with control (Figs. 6D and S8B), without any change of mitochondrial mass and mitochondrial superoxide, suggesting higher energy production.

Next, we explored the possibility of greater efficacy in relapse prevention by combining ATO-induced ROS production with a decrease in antioxidant defenses, based on previous studies suggesting that redox combination therapy is a clinically promising approach in the treatment of advanced solid tumors [41]. We chose to combine ATO with buthionine sulfoximine (BSO), an inhibitor of glutathione synthesis, which also leads to increased ROS levels, including in PDAC cells, but has no antitumor activity when used alone [41, 42, 51]. BSO treatment combined with ATO was also performed for 2 months in both xenografts. Importantly, we observed that the combination of ATO and BSO completely prevented relapse in both PDAC xenografts (Fig. 6E, F). We did not observe any relapse during the three months following the end of the combined treatment (we sacrificed the mice at this time).

Collectively, these data suggest that the combination of ROS overproduction (ATO) and inhibition of antioxidant capacity (BSO) might be a promising strategy to prevent PDAC relapse.

## Discussion

In this work, we demonstrate that PDAC disease relapse in vivo happens through tumor growth from drug-tolerant persister (DTP) cancer cells. As relapse occurs after a drug holiday in our experimental setting, we now use the term tolerance instead of resistance (defined as the tumor’s ability to grow under treatment) [15]. We show that the PDAC DTP cells survive during treatment-induced tumor regression due to redox metabolic adaptations (schematic model in Fig. S9). The existence of DTP cells in tumors was demonstrated during the last decade, and their major biological features were defined: lack of additional genomic alteration, stagnant cell proliferation, reversible drug sensitivity, and flexible energy metabolism [12, 13, 15, 52, 53]. These cells are difficult to study due to their low number in the minimal residual disease (scar of the regressed tumor) and lack of specific marker. This is why we analyzed tumors under treatment just before total regression and at the onset of tumor relapse, finding the same metabolic characteristics as in recurrent tumors with bigger volume at the ethical endpoint.

Regarding the reversible drug-sensitivity property of DTP cells, we had shown previously that the PDAC084T xenograft model used throughout this study responded to a second cycle of treatment after relapse [32]. With regard to the characteristic of flexible energy metabolism, we show here that DTP PDAC cells exhibit higher mitochondrial oxidative metabolism than PDAC cells from untreated mice (increased mitochondrial mass, active polarized mitochondria, increased ATP production, high ROS and antioxidant levels). These characteristics have been observed in several different cancers, such as melanoma, acute myeloid leukemia, colorectal, and breast cancer [13]. The recent literature is consistent with our data by reporting chemotherapy-induced increase in OXPHOS and antioxidants in PDAC cells, which play a role in primary resistance and acquired tolerance [54–56].

In addition to the enrichment of the redox pathway in relapsed tumors, our RNA-seq transcriptomic analysis reveals other pathways that are also features of DTP cells. Rewiring of lipid metabolism has been shown to promote cell viability and to be an actionable vulnerability [15]. EMT, which is induced by ROS and hypoxia in DTPs, correlates with their phenotypic plasticity as well as heterogeneity within the DTP population [15]. Interestingly, the transcription factor Zeb1 was reported to have a substantial role in EMT activation, promoting cell plasticity and metastasis in pancreatic cancer, as well as in ferroptosis, a cell death pathway induced by oxidized lipids [57, 58]. Hypoxia, MAPK signaling pathway, extracellular matrix (ECM), and inflammation are major microenvironmental cues supporting the DTP phenotype [15]. Finally, the cAMP signaling pathway was shown to mediate adaptive mitochondrial stress response in DTP AML cells [59].

Importantly, this work is the first demonstration of the existence of PDAC DTP cells in vivo. The experimental background in the pioneer study of Viale et al. in 2014 [28] was that of cells surviving genetic ablation of the Kras oncogene responsible for tumor relapse and relying on mitochondrial respiration to survive, whereas our experimental model implementing drug treatments is a true context of persistence supported by drug tolerance.

New therapies that effectively eradicate primary resistant cancer cells and residual cells after a therapeutic response are an urgent medical need. A better knowledge of the mechanisms of therapy-induced drug tolerance is required to develop strategies to prevent the emergence of drug-tolerant cells or to eradicate them to prevent tumor relapse [15, 22, 48, 52]. Targeting mitochondrial metabolic adaptations during treatment, as in chronic myeloid leukemia [18], could not be used in PDAC because these adaptations also occur in tumors of our xenograft model treated with gemcitabine combined with perhexiline, which inhibits mitochondrial activity. In contrast, we show that targeting the adaptations in redox metabolism at the MRD period is a promising therapeutic approach for preventing relapse in PDAC. We have exploited the increased ROS and antioxidant content in DTP cells, which promote cell survival, to implement a strategy aimed at inducing severe oxidative stress likely to promote DTP cell eradication. This was achieved by the use of drugs that further increase intracellular ROS levels, combining increased ROS production with a pro-oxidant and disruption of the cellular antioxidant system [22, 60].

In addition to drugs targeting mitochondrial oxidative metabolism, there are other metabolic or cell death pathways that are targetable in DTP PDAC cells, such as glutamine metabolism and ferroptosis [61–64]. These latter studies have the same limitations as ours, namely that they lack a comprehensive molecular analysis of DTP cancer cells at the single-cell level, as well as a spatial study, to consider cellular heterogeneity in PDAC tumors. Considering the importance of tumor microenvironment immune cells in the antitumoral response and DTP clearance, as well as the central role of metabolism in immune cell activity and fate [13, 15], we propose that all new therapeutic strategies be implemented in immunocompetent preclinical models. Our prospects are to continue this study of PDAC DTP cancer cells using only the immunocompetent relapse model, to assess the microenvironmental mechanisms underlying DTPs immune evasion, and develop new treatment combinations for therapeutic success.

In conclusion, the strength of our study is the demonstration for the first time of PDAC DTP cells in vivo (and not in vitro), in two different models of PDAC mice, either immunodeficient (heterotopic xenografts) or immunocompetent (orthotopic syngeneic allografts), both responding to chemotherapeutic treatment but relapsing after complete regression. Furthermore, we illustrate for the first time the possibility of preventing tumor growth relapse from DTP cells by inducing their eradication through strong oxidative stress. Collectively, combining the promotion of ROS production and the inhibition of antioxidant capacity could be a promising avenue for treating pancreatic cancer in the clinic.

## Supplementary information

Supplementary information

Dataset 1

Dataset 2

## Data Availability

All data, including RNA-seq data, were deposited in Mendeley public repository, Reserved DOI: 10.17632/z6pb8sbxy2.1, and will be made public after acceptance for publication.
