# Supplementary material for: Adaptation of redox metabolism in drug-tolerant persister cells is a vulnerability to prevent relapse in pancreatic cancer
Source: Oncogenesis. 2025 Dec 9;14(1):48. doi: 10.1038/s41389-025-00591-0 (PMC12690124; doi:10.1038/s41389-025-00591-0)
Supplement: Supplementary file 1 — Supplementary information [file 41389_2025_591_MOESM1_ESM.pdf]

SUPPLEMENTARY INFORMATION

Adaptation of redox metabolism in drug-tolerant persister cells is a vulnerability to prevent relapse in pancreatic cancer

Nadine Abdel Hadi[1], Gabriela Reyes-Castellanos[1], Tristan Gicquel[1], Scarlett Gallardo-Arriaga[1], Emma Cosialls[1], Emeline Boet[2], Jean-Emmanuel Sarry[2], Rawand Masoud[1], Juan Iovanna[1], Alice Carrier[1,*]

[1]Aix Marseille Univ, Inserm, CNRS, Institut Paoli-Calmettes, Centre de Recherche en Cancérologie de Marseille (CRCM), Marseille, France.

[2]Centre de Recherches en Cancérologie de Toulouse, Université de Toulouse, Inserm, CNRS, Toulouse, France.

*Corresponding author, alice.carrier@inserm.fr.

Supplementary Figures: 9

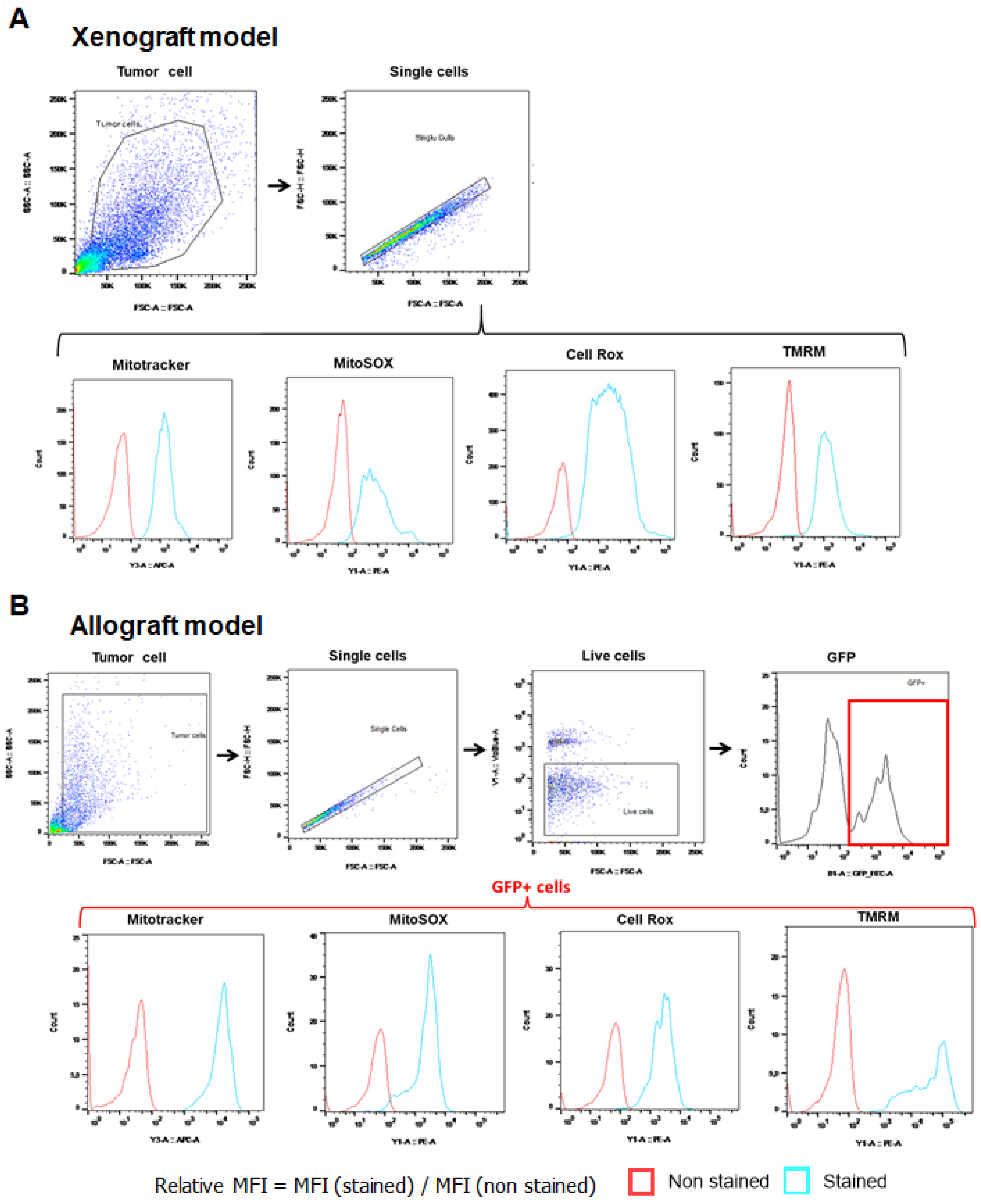

Figure S1. Representative flow cytometry plots of tumor cell populations from xenograft and allograft mouse models (related to Figures 1 and 2). (A) In xenograft model, tumor cells are separated from debris by Forward Scatter Area (FSC-A)/Side Scatter Area (SSC-A), and doublets are excluded by FSC-A and FSC-height (FSC-H) dot plots. Mitotracker Deep Red, MitoSOX Red, CellROX Orange, and TMRM histograms are done by gating on single cells**. (B)** In allograft model, the histograms are done by gating on GFP^+ cells (KPCluc2 cancer cells) after gating on live cells (the negative fraction for VioBlue-A staining).

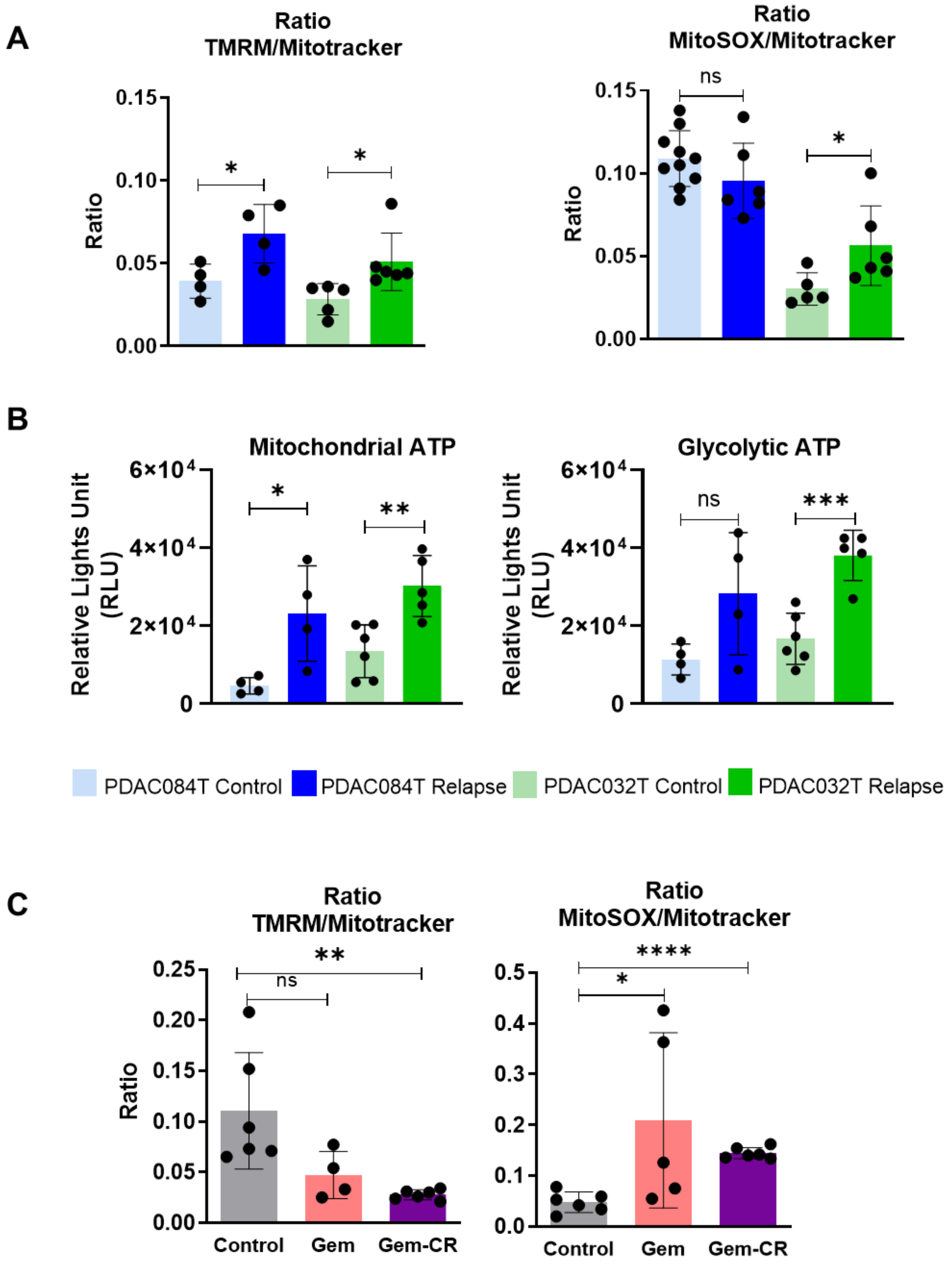

Figure S2. Mitochondrial and redox metabolic reprogramming in relapsed PDAC xenografts and allografts (related to Figures 1 and 2). TMRM (measuring mitochondrial membrane potential) and MitoSOX (measuring mitochondrial superoxide anions) values were normalized by Mitotracker (measuring mitochondrial mass) values in relapsed xenografts (**A**), and relapsed allografts (**C**). **(B)** ATP production was measured in the presence of oligomycin (left) or 2DG (right) *in vitro* on dissociated relapsed xenografts, allowing to calculate mitochondrial and glycolytic ATP percentages shown in Figure 1D.

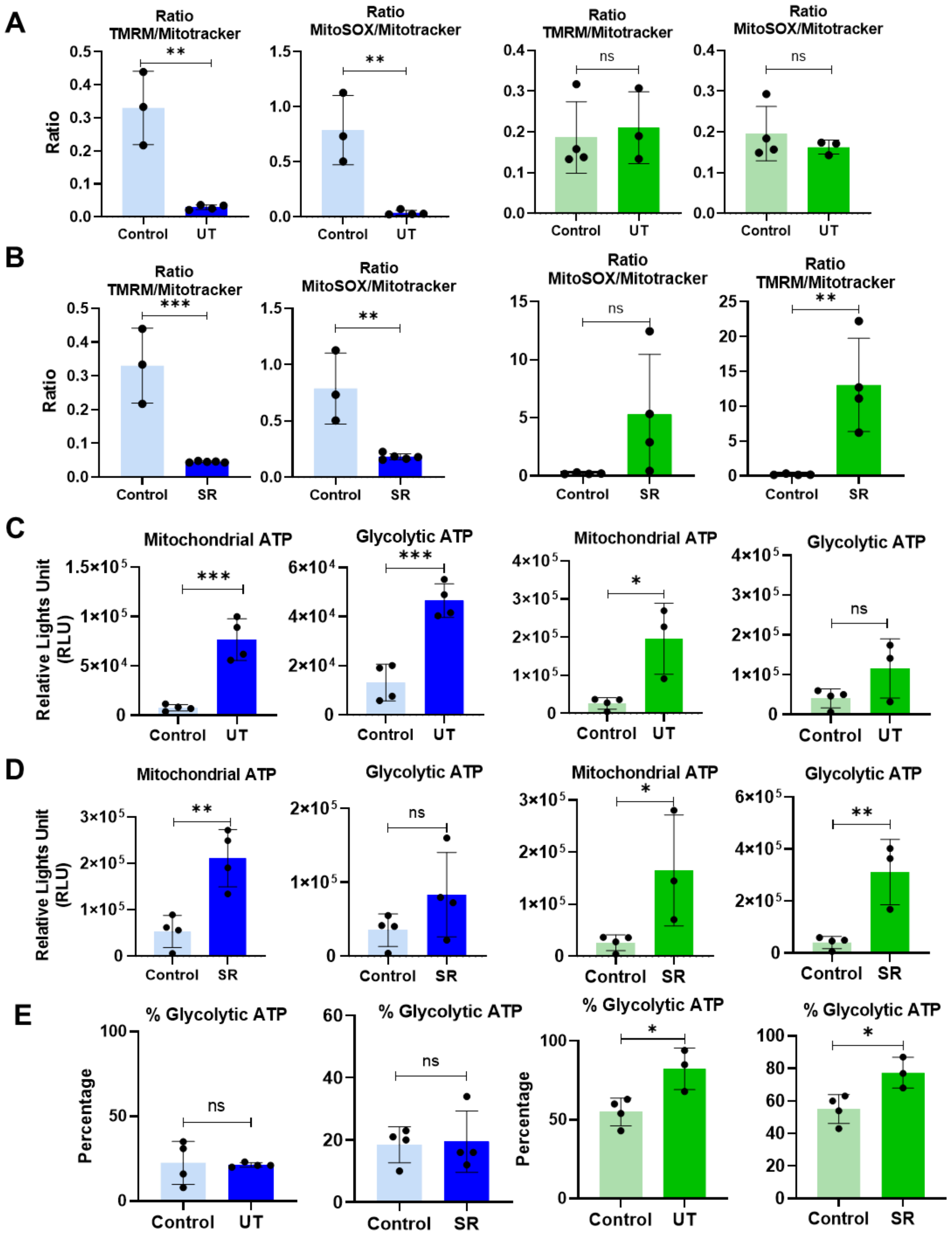

Figure S3. Mitochondrial and redox metabolic reprogramming occurs during treatment-induced complete regression in PDAC xenografts (related to Figure 3). (A-B) TMRM and MitoSOX values were normalized by Mitotracker values in xenografts PDAC084T (left) and PDAC032T (right), under treatment (UT, **A**) and at start of relapse (SR, **B**). **(C-D)** ATP production was measured in the presence of oligomycin or 2DG *in vitro* on dissociated relapsed xenografts PDAC084T (left) and PDAC032T (right), under treatment (UT, **C**) and at start of relapse (SR, **D**). **(E)** Percentages of glycolytic ATP in PDAC084T (left) and PDAC032T (right), under treatment (UT) and at start of relapse (SR).

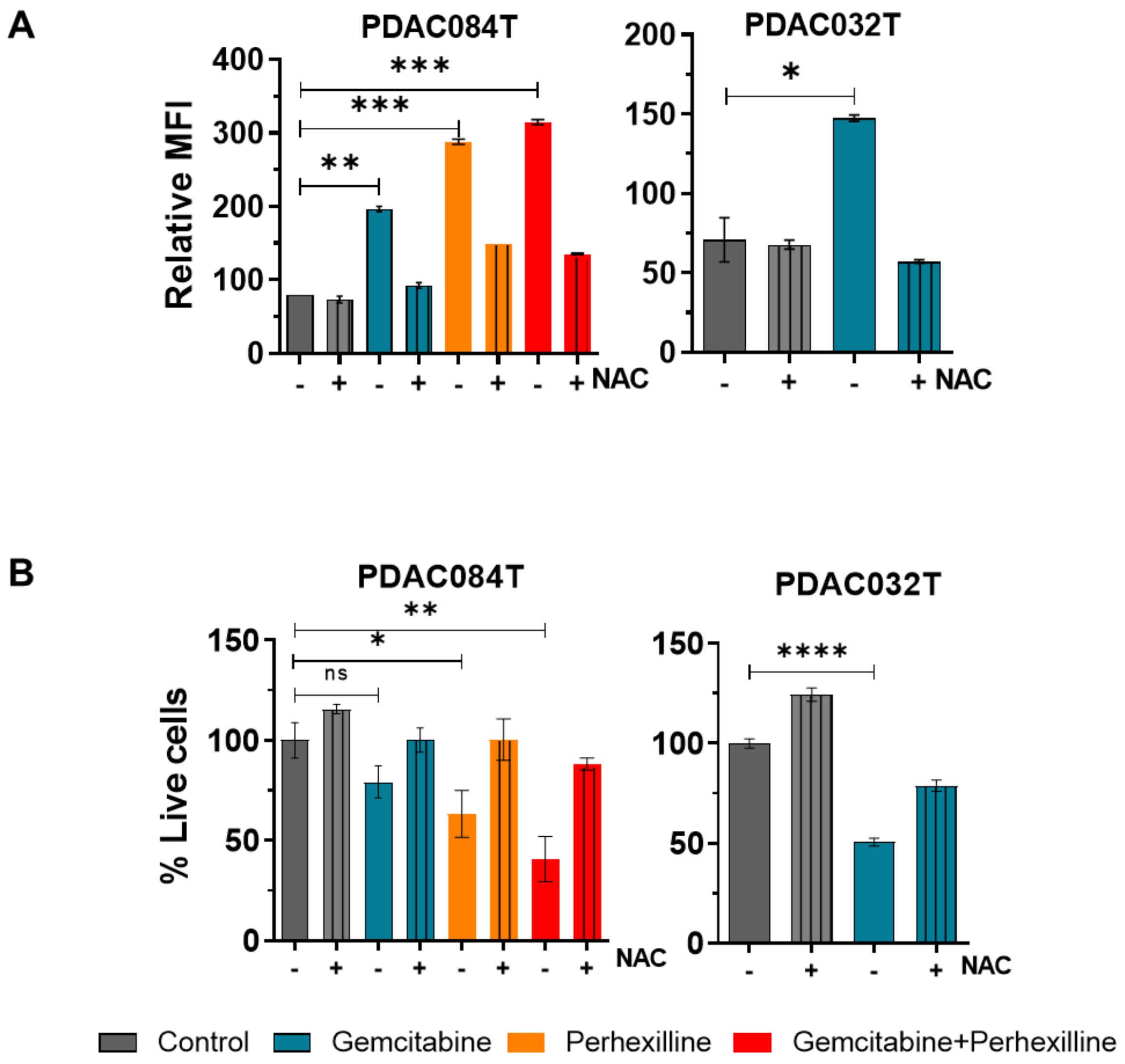

Figure S4. Chemotherapy induces ROS increase and redox-driven loss of cell viability in PDAC cells *in vitro* (related to Figure 3). (A) Total ROS level was measured by flow cytometry with the CellROX orange probe in PDAC084T and PDAC032T cells cultured *in vitro*, after 24 hours treatment with gemcitabine (1 µM), or perhexiline (7 µM), or combination at same concentrations. We used DMSO (0.05%) as vehicle for the controls. Supplementation with the antioxidant N-acetylcysteine (NAC) at 2.5 mM was performed for 24 hours at the same time as the treatments. Data are expressed as mean of duplicates ± SEM and are representative of three independent experiments. To calculate p values, Unpaired T-test was used. **(B)** Percentage of live cells treated for 24 hours with gemcitabine (1 µM), perhexiline (7 µM) or the combination in PDAC084T, and with gemcitabine alone in PDAC032T. Cell viability was determined by Crystal violet assay. Data are means of triplicates ± SEM, and are representative of three independent experiments. Unpaired T-test was used. *, **, *** and **** correspond to $p<0.05$, 0.01, 0.001, and 0.0001, respectively; ns: non-significant difference.

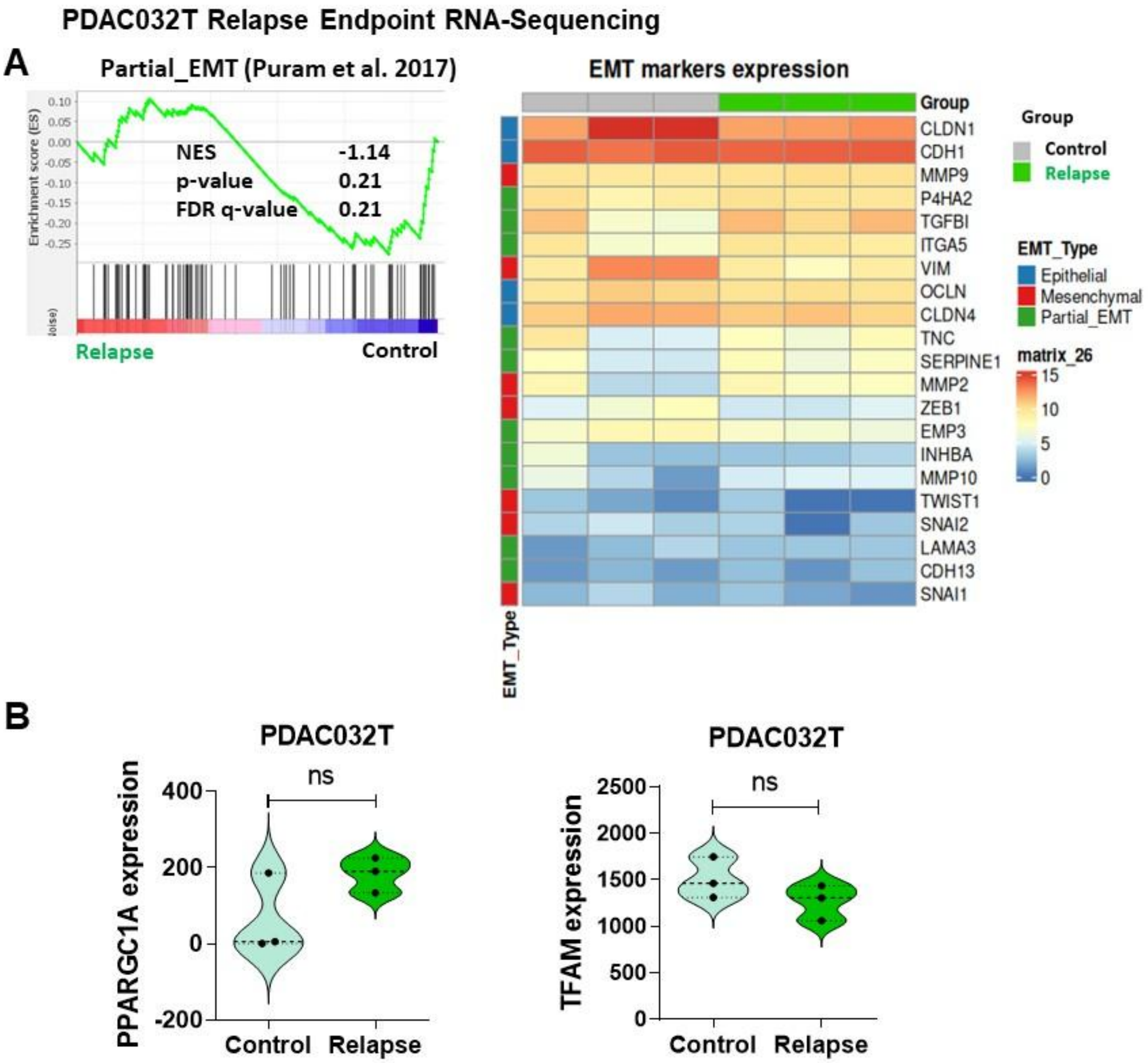

Figure S5 (related to Figure 4). (A) GSEA using the partial EMT signature from Puram et al. 2017 (Left), and heatmap showing EMT markers expression (Right), comparing RNA sequencing data from PDAC032T relapsed tumors with control. **(B)** *PPARGC1A* and *TFAM* expression in PDAC032T relapsed tumors versus control based on RNA sequencing data. ns, not significant.

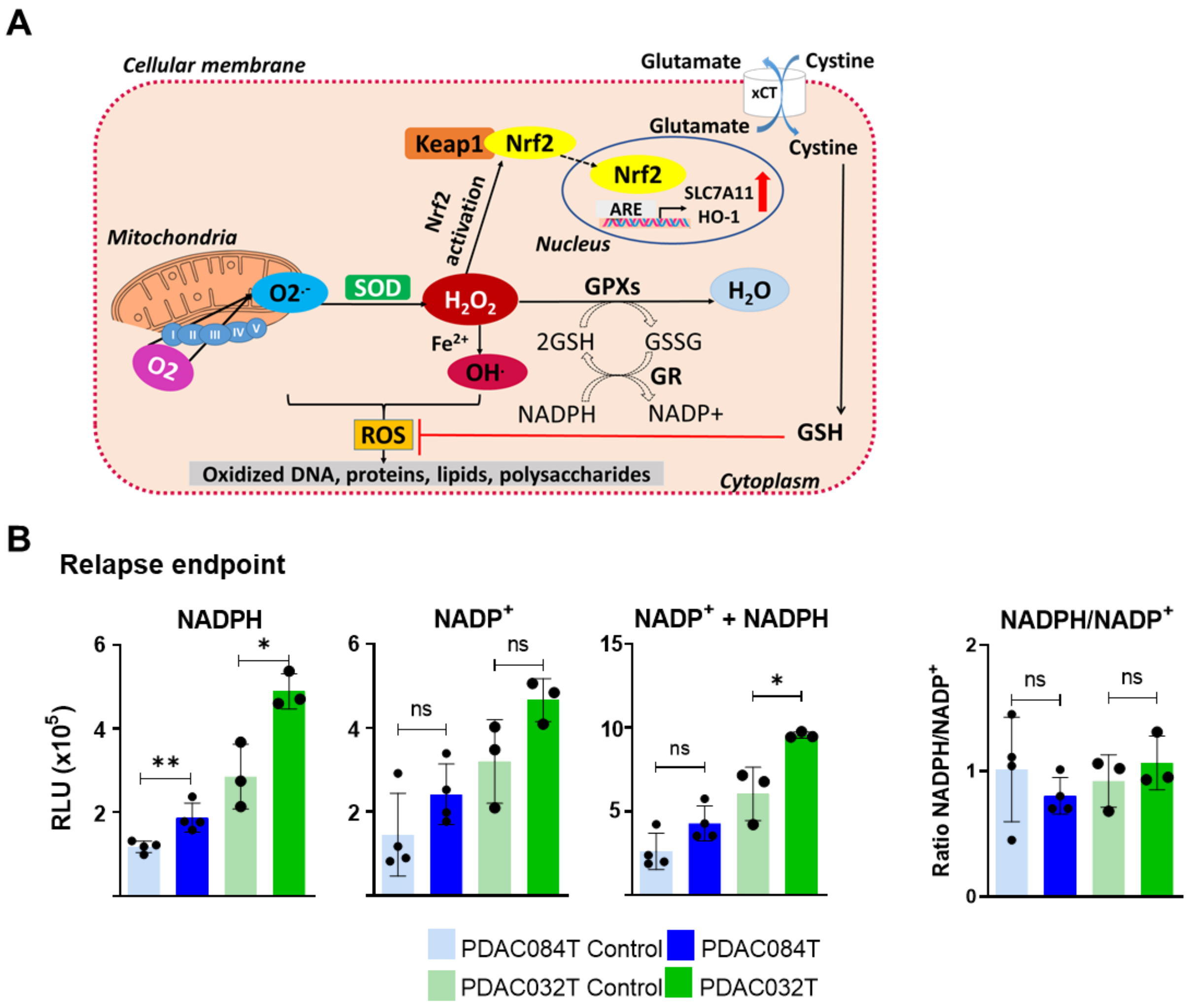

Figure S6. Antioxidant defenses in xenografts at relapse end point (related to Figure 5). (A) Simplified schematic representation of ROS generation and enzymatic and non- enzymatic antioxidant defenses. Superoxide anion ($O_2^{\cdot-}$) is mainly produced by the mitochondrial ETC complexes by partial reduction of molecular oxygen (O_2). Superoxide is dismutated into hydrogen peroxide (H_2O_2) by superoxide dismutases (SOD). H_2O_2 is converted to water (H_2O) by glutathione peroxidases (GPX). Via the Fenton reaction with metal ions Fe^{2+} or Cu^{+}, H_2O_2 is further reduced to highly reactive hydroxyl radical ($OH^{\cdot}$), thereby damaging biological macromolecules such as DNA, lipids, and proteins. H_2O_2 is the main player in redox homeostasis, and can induce the activation of the antioxidant transcription factor Nrf2 through dissociation of the Nrf2-KEAP1 complex, phosphorylation of Nrf2, and its nuclear translocation. In the nucleus, Nrf2 promotes transcription of multiple antioxidant genes such as *SLC7A11* and *HO-1* by binding to the antioxidant responsive elements (ARE) in the promoter region of target genes. Via its entry into cells through the glutamate/cystine antiporter xCT encoded by the *SLC7A11* gene, cysteine can enhance GSH production which is a tripeptide glutamate-cysteine-glycine. The antioxidant function of GSH is mediated by two enzymes: GPX and GR. GPX allows the reduction of H_2O_2 by the oxidation of GSH (reduced glutathione) to GSSG (oxidized glutathione). The GSSG is subsequently reduced to GSH by GR at the expense of NADPH used as a cofactor. **(B)** NADPH, $NADP^{+}$ (oxidized form of NADPH), and total $NADP^{+}$ + NADPH levels were measured using the NADP/NADPH-Glo™ Assay kit, and the ratio NADPH/$NADP^{+}$ was calculated.

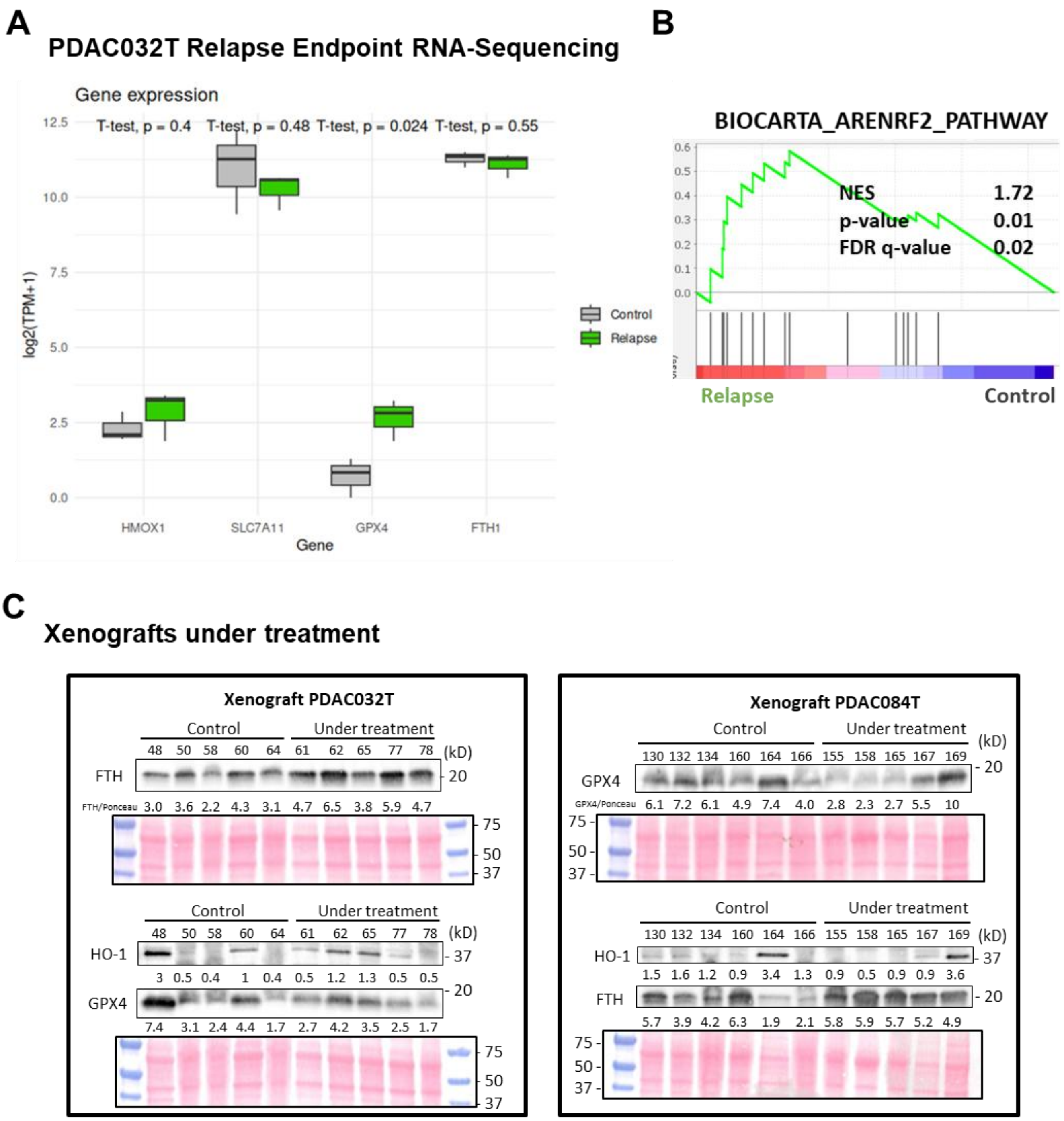

Figure S7 (related to Figure 4 and 5). (A) Nrf2 target gene (*HMOX1* is encoding HO-1) and *GPX-4* expression in the RNA-seq data from PDAC032T relapsed tumors and control tumors. *NRF2* mRNA was not detected in these data. **(B)** GSEA using the BIOCARTA_ARENRF2 pathway comparing RNA seq data from PDAC032T relapsed tumors with control. **(C)** Immunoblotting analysis of GPX4 protein and two Nrf2 target proteins, HO-1 and ferritin heavy chain (FTH), in both xenograft models during treatment. The ratios protein/ponceau red are shown.

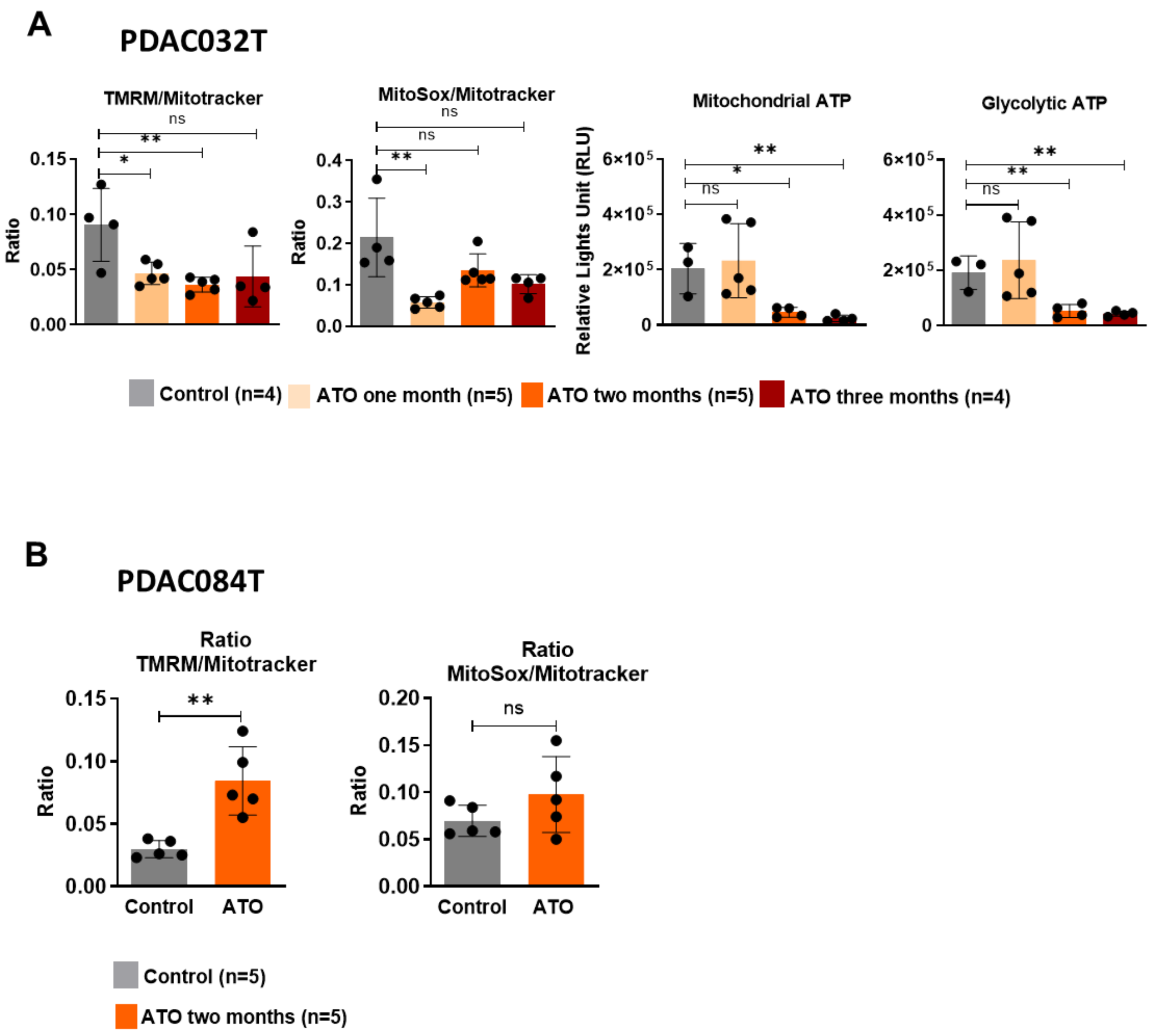

Figure S8. Mitochondrial metabolic reprogramming during ATO treatment in PDAC xenografts (related to Figure 6). (A-B) TMRM and MitoSOX values were normalized by Mitotracker values in xenografts PDAC032T (**A, left**) and PDAC084T (**B**), and ATP production was measured in the presence of oligomycin or 2DG *ex vivo* on dissociated PDAC032T xenografts (**A, Right**).

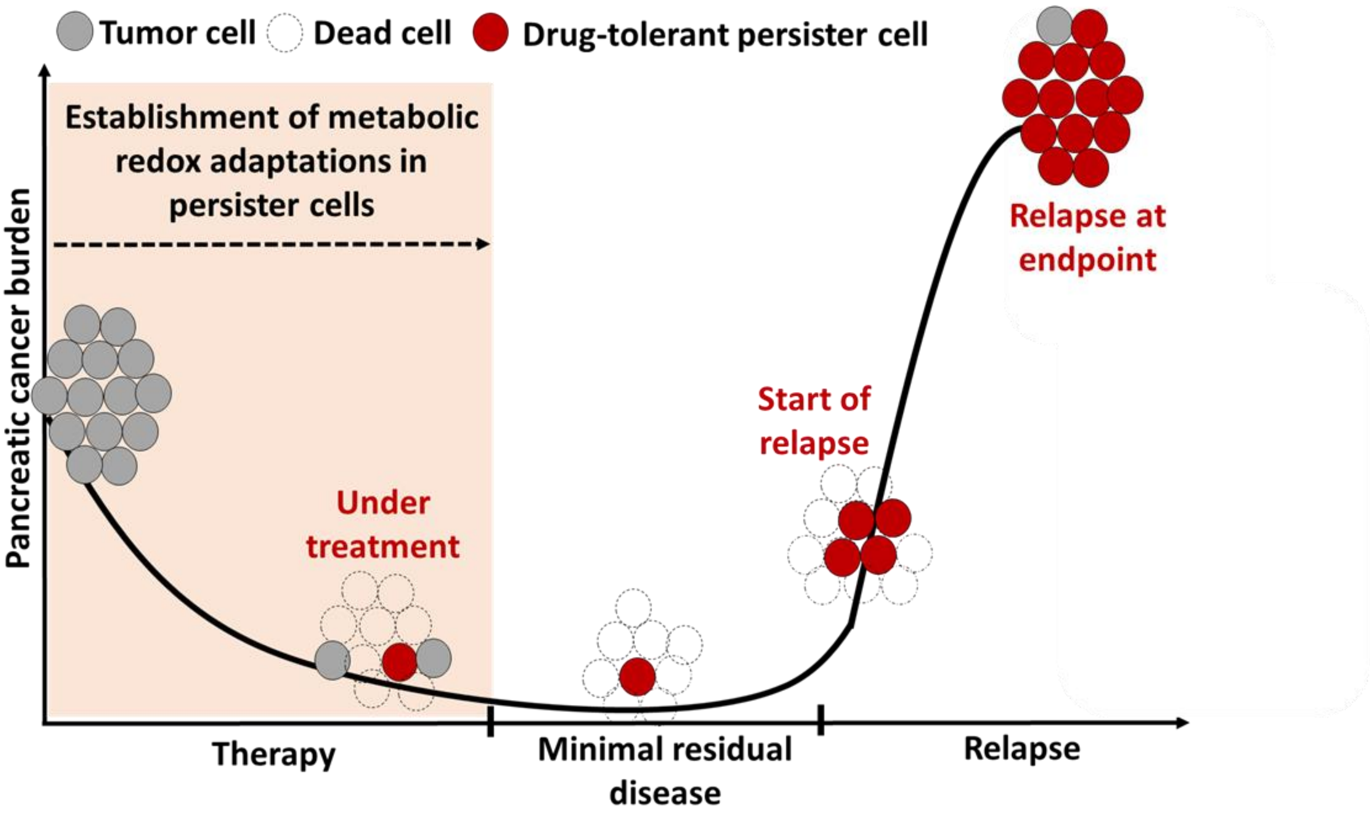

Figure S9. Working model of treatment-induced acquired tolerance in PDAC. In tumors that respond to therapy, most tumor cells die (dead cells) during treatment, ensuring therapy-induced regression. However, some drug-tolerant persister (DTP) cancer cells survive during regression, through the establishment of metabolic (mitochondrial and redox) adaptations. They are maintained in the tumor scar and are associated with what is known as minimal residual disease. When these DTP cancer cells resume their proliferation, they are at the origin of the tumor regrowth and disease relapse. Metabolic changes are observed in DTP cells at all stages analyzed and not just in the tumor after relapse, supporting that they are induced by therapeutic treatment and not by tumor growth after relapse.
