# Supplementary material for: Adaptation of redox metabolism in drug-tolerant persister cells is a vulnerability to prevent relapse in pancreatic cancer
Source: Oncogenesis. 2025 Dec 9;14(1):48. doi: 10.1038/s41389-025-00591-0 (PMC12690124; doi:10.1038/s41389-025-00591-0)

Original data Immunoblots Figure S7C Xenograft PDAC032T

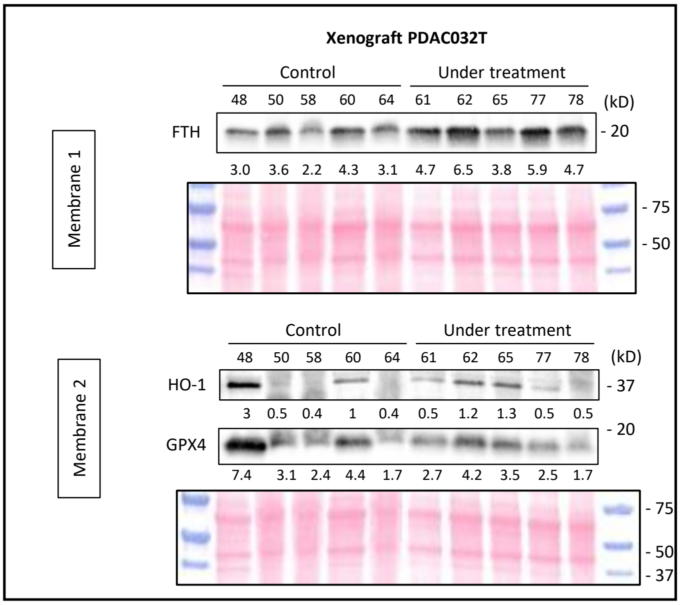

Membrane 1

FTH
22 kD

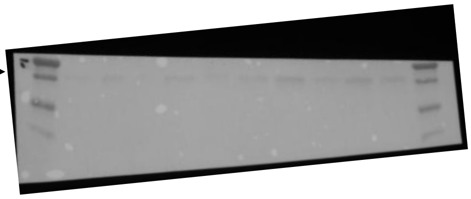

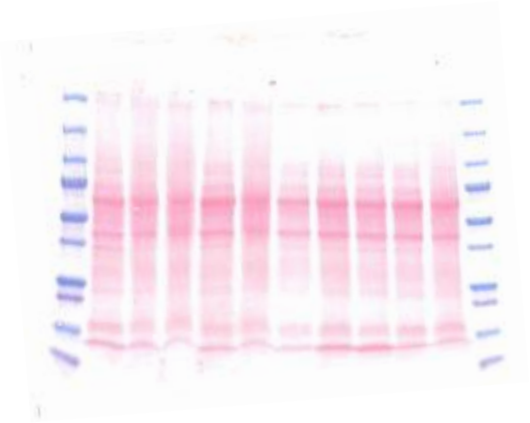

Membrane 2

HO-1
33 kD

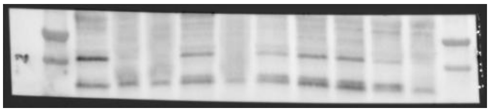

GPX-4
20 kD

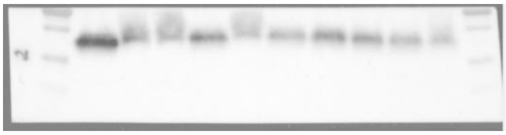

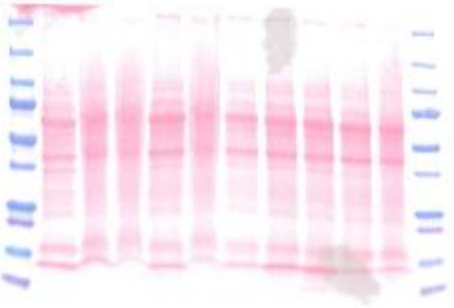

Original data Immunoblots Figure S7C Xenograft PDAC084T

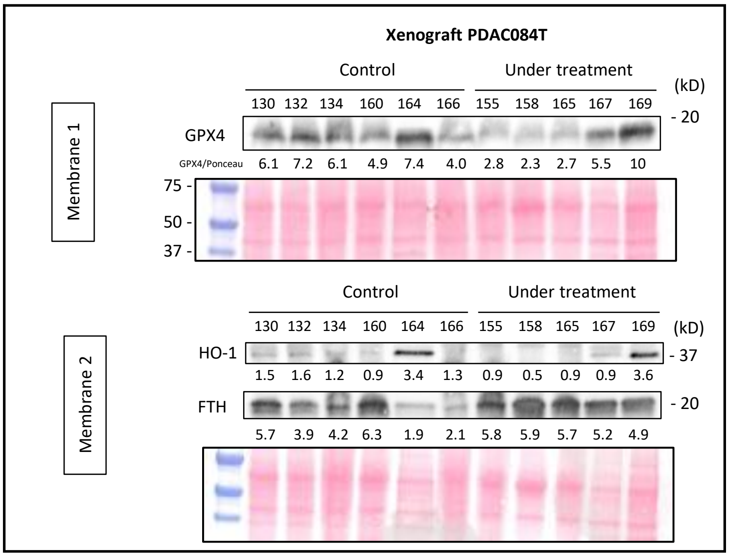

Membrane 1

GPX-4
20 kD

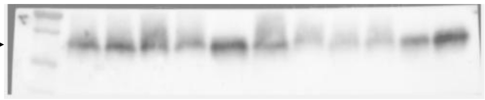

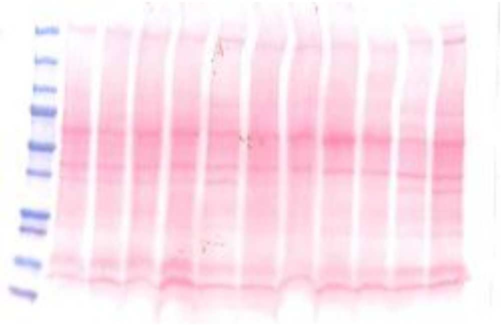

Membrane 2

HO-1
33 kD

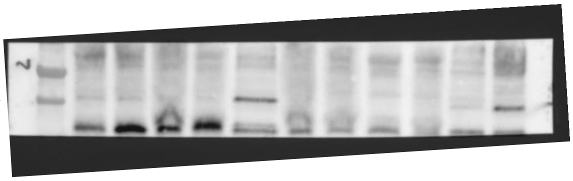

FTH
22 kD

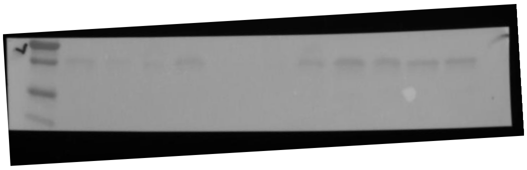

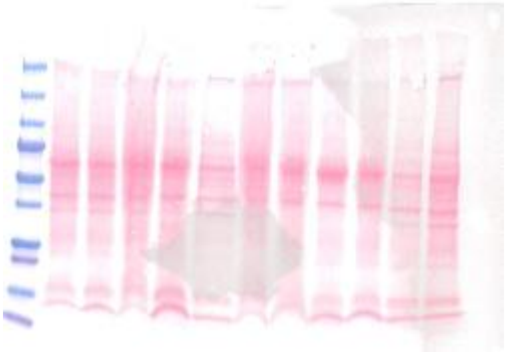

Supplement: Supplementary file 2 — Dataset 1 [file 41389_2025_591_MOESM2_ESM.pdf]
